# Anti-tumor effects of ONC201 in combination with VEGF-inhibitors significantly impacts colorectal cancer growth and survival in vivo through complementary non-overlapping mechanisms

**DOI:** 10.1186/s13046-018-0671-0

**Published:** 2018-01-22

**Authors:** Jessica Wagner, C. Leah Kline, Lanlan Zhou, Vladimir Khazak, Wafik S. El-Deiry

**Affiliations:** 1 Laboratory of Translational Oncology and Experimental Cancer Therapeutics, Molecular Therapeutics Program and Department of Hematology/Oncology, Fox Chase Cancer Center, Philadelphia, PA USA; 2 NexusPharma, Inc., Philadelphia, PA USA

**Keywords:** ONC201, Bevacizumab, Cancer therapy

## Abstract

**Background:**

Small molecule ONC201 is an investigational anti-tumor agent that upregulates intra-tumoral TRAIL expression and the integrated stress response pathway. A Phase I clinical trial using ONC201 therapy in advanced cancer patients has been completed and the drug has progressed into Phase II trials in several cancer types. Colorectal cancer (CRC) remains one of the leading causes of cancer worldwide and metastatic disease has a poor prognosis. Clinical trials in CRC and other tumor types have demonstrated that therapeutics targeting the vascular endothelial growth factor (VEGF) pathway, such as bevacizumab, are effective in combination with certain chemotherapeutic agents.

**Methods:**

We investigated the potential combination of VEGF inhibitors such as bevacizumab and its murine-counterpart; along with other anti-angiogenic agents and ONC201 in both CRC xenograft and patient-derived xenograft (PDX) models. We utilized non-invasive imaging and immunohistochemistry to determine potential mechanisms of action.

**Results:**

Our results demonstrate significant tumor regression or complete tumor ablation in human xenografts with the combination of ONC201 with bevacizumab, and in syngeneic MC38 colorectal cancer xenografts using a murine VEGF-A inhibitor. Imaging demonstrated the impact of this combination on decreasing tumor growth and tumor metastasis. Our results indicate that ONC201 and anti-angiogenic agents act through distinct mechanisms while increasing tumor cell death and inhibiting proliferation.

**Conclusion:**

With the use of both a murine VEGF inhibitor in syngeneic models, and bevacizumab in human cell line-derived xenografts, we demonstrate that ONC201 in combination with anti-angiogenic therapies such as bevacizumab represents a promising approach for further testing in the clinic for the treatment of CRC.

**Electronic supplementary material:**

The online version of this article (10.1186/s13046-018-0671-0) contains supplementary material, which is available to authorized users.

## Background

Colorectal cancer is the third most common cancer in the world and has an overall 5-year survival rate of approximately 10% for those with stage IV cancer [1, 2]. The cure rate and 5-year survival rate is significantly lower for those with metastatic colorectal cancer who cannot be cured from surgery alone. Therefore, therapies that can treat metastatic colorectal cancer is needed [3].

The novel anti-cancer imipridone ONC201 induces the *TRAIL* and *DR5* genes through dual inactivation of Akt/ERK/Foxo3a and activation of the integrated stress response (ISR). Further, in vivo, ONC201 possesses a broad spectrum of activity, wide safety margin, robust stability, aqueous solubility, and favorable pharmacokinetics [4–13]. The therapeutic activity of ONC201 in preclinical in vivo studies in solid tumors, hematological malignancies, and with targeting of cancer stem cells as well as bulk tumor cells prompted its ongoing clinical development. In Phase I clinical testing with ONC201, patients were treated with the compound once every 3 weeks and the drug showed evidence of safety and promising efficacy in multiple tumor types [14].

Tumor angiogenesis is the process by which new blood vessels are developed; a critical process in tumor progression and development [15]. Many growth factors are needed for angiogenesis including vascular-endothelial growth factor (VEGF), fibroblast growth factors, and platelet-derived endothelial growth factors, which bind to three tyrosine kinase receptors: VEGFR1/2 which promote angiogenesis, and VEGFR3 which stimulates lymphangiogenesis [16]. These corresponding receptors are located on endothelial cells of pre-existing blood vessels and promote the activation of endothelial cells [17].

High levels of VEGF has been shown to increase vascular disorganization and permeability; creating heavily leaky tumors with poor perfusion and enhancing the ability of tumor cells to spread throughout the body [18]. Further, higher VEGF expression levels has been detected in various human cancers including colorectal and non-small lung cancer and have some correlation to outcome [19–21].

Bevacizumab (Avastin), a humanized monoclonal antibody designed to neutralize human VEGF, inhibits VEGF-induced proliferation of endothelial cells and promotes endothelial cell apoptosis. Treatment with monoclonal antibodies such as bevacizumab have been show to inhibit growth of tumors in vivo, promote tumor cell apoptosis, and prevent the spread of metastases [22–25]. Bevacizumab functions best as a combinational agent and has shown promise in combination with several approved chemotherapies including with 5-fluorouracil or paclitaxel; causing it to be approved by FDA for metastatic CRC, non-small cell lung cancer, and metastatic breast cancer [22, 26–28]. Regorafenib, an oral multi-kinase inhibitor with anti-angiogenic properties is also approved for metastastic CRC but has a distinct profile of adverse advents including hepatotoxicity, fatigue, diarrhea, hypertension, and hand-foot syndrome [29, 30]. Here we demonstrate that ONC201 and bevacizumab, or its murine counterpart, provide a potent combinational therapy option when compared to regorafenib that could be further pursued in the clinic.

## Methods

### Cell lines and PDX tumors

All cell lines were obtained from the American Type Culture Collection. CT26 and MC38 cells were provided by Dr. Scott Waldman’s lab at Thomas Jefferson University. ONC201 was provided by Oncoceutics.

The PDX tumor was provided by NexusPharma Inc., Philadelphia, PA. The PNX0229 sample was obtained from a 57-year old Caucasian male with a Stage 2A descending colon adenocarcinoma. The sample was taken from a liver metastases that formed. The patient underwent a combination of FOLFIRI and Erbitux with a partial response; and a second line therapy of FOLFOX with progressive disease before the resection.

### Small molecules and dosing schedule

ONC201 was administered orally in 10:70:20 DMSO:PBS:Cremphor El as described [4] and treated weekly at the indicated doses. Bevacizumab was procured from the Fox Chase Cancer Center pharmacy and diluted in PBS. Bevacizumab was administered through retro-orbital injections every other week at a dose of 5 mg/kg. Regorafenib was procured from MedChemExpress (HY-1031) and administered orally at 10 mg/kg per day dissolved in PBS for at least 22 days. Anti-murine VEGF-A inhibitory antibody (Biolegend 512,808) was administered at 10 micrograms by i.p. twice weekly. Mouse body weight was observed every 3 days and when body weight began to drop regorafenib dosing was stopped.

### In vivo animal experiments

All animal experiments were conducted in accordance with the Institutional Animal Care and Use Committee at Fox Chase Cancer Center. For subcutaneous xenografts, 6-week-old female athymic nu/nu mice (Taconic Biosciences) were inoculated with 1 × 10^6^ cells of the HT29-luciferase, HCT116 *p53*^*−/−*^, or HCT116-GFP cell lines in each rear flank, in a 150 μl suspension of 1:1 Matrigel (BD). For subcutaneous xenografts in syngeneic models, CT26 cells were inoculated with 1.0 × 10^6^ cells into six-week old female Balb/c mice (Taconic Biosciences) and MC38 cells were inoculated with 1.0 × 10^6^ cells into six-week old female C57/BL6 mice (Taconic Biosciences). All subcutaneous tumors were allowed to establish for 1 to 3 weeks after injection until they reached a volume of ~200 mm^3^ before treatment initiation. Mice were monitored every 3 days and tumor volumes were measured using calipers. Tumor volumes were measured according to the formula (L*W^2)/2.

### In vivo pathology and toxicology

Toxicity during the course of ONC201 and combination treatment was judged by body weight decrease of greater than 10%, tumor growth of more than 10% of body weight, or a body condition scoring < 2. Serum and plasma samples were collected through orbital bleeding and cardiac puncture before sacrifice, and samples were immediately stored at 4 °C and processed by Antech Diagnostics for CBC and chemistry panels. Results were analyzed by board-certified toxicologists. Tumors were measured post-mortem through caliper and water density examination. Organ and tumor samples were processed in 10% formalin and fixed in paraffin. Hematoxylin-stained samples were analyzed by a board-certified pathologist to determine whether tumor cells existed on any organs or necrosis occurred in tumors. Board-certified veterinary pathologists also indicated whether or not signs of toxicity were present.

### Immunohistochemistry

All antibodies were purchased from Cell Signaling. After fixation, the tumor samples were embedded in paraffin and 8 μm sections were cut and mounted on slides. The sections were then processed and analyzed using immunohistochemistry with VEGF, Ki67, and CD31 [4]. CD31 was only scored on those tumors +/− 600 mm^3^ from the average tumor size in order to best control for tumor size variation. CD31, and Ki67 levels where calculated by independent blind-scoring and the use of VECTRA 3.0 Automated Quantitative Pathology Imaging system and Inform 2.0 software cursory of the Fox Chase Cancer Center Biosample Repository.

### HUVEC assays

Human normal primary umbilical vein endothelial cells where purchased from ATCC and HUVEC assay was used following the protocol from Millipore (ECM625). Imaging occurred at 10 h unless indicated. IC50 doses where used as previously published. Sprouting was counted and calculated as described in Millipore methods.

### Fluorescent probe imaging

Mice where anesthetized with isoflurane and injected through retro-orbital *i.v.* injections at recommended amounts for either Superhance 680 (NEV10116) or Angiosense 750 (NEV10011EX). Mice were allowed to equilibrate for the recommended times for each probe; and then they were imaged as directed by Perkin Elmer on a Maestro in-vivo imaging system (Cri, Woburn, MA). Mice with Superhance were also imaged for GFP-expressing tumors. Relative luminescence units (RLU) where calculated by measuring tumor fluorescence subtracted from the background of mouse auto-fluorescence divided by the area of tumor, measured by ImageJ.

### ELISA assays

A total of 500 μL blood was collected through orbital blood draw while mice where anesthetized and plasma was collected in EDTA tubes and serum collected in Heparin separating tubes. Tubes were spun at 1000 rpm for 15 min. Samples were analyzed using Human TRAIL/TNSFSF10 Quantikine ELISA kit (R&D Systems, Minneapolis MN) or Human Apoptosense M30 ELISA kit (MyBiosource MBS9300524). All analyses were performed according to the manufacturer’s directions.

### Statistical analysis

Data are presented as means + SD. To assess the statistical significance of the differences, unpaired Student’s *t* test with Holm-Sidak correction for multiple comparisons (maximum of three comparisons were made) was performed, with *P* < 0.05 deemed statistically significant. Measurements from three biological replicates per treatment group were compared. Unless otherwise noted in the figure legend, comparisons were made against the vehicle control.

## Results

### ONC201 shows combinatorial efficacy with bevacizumab in vivo

**Fig. 1:**
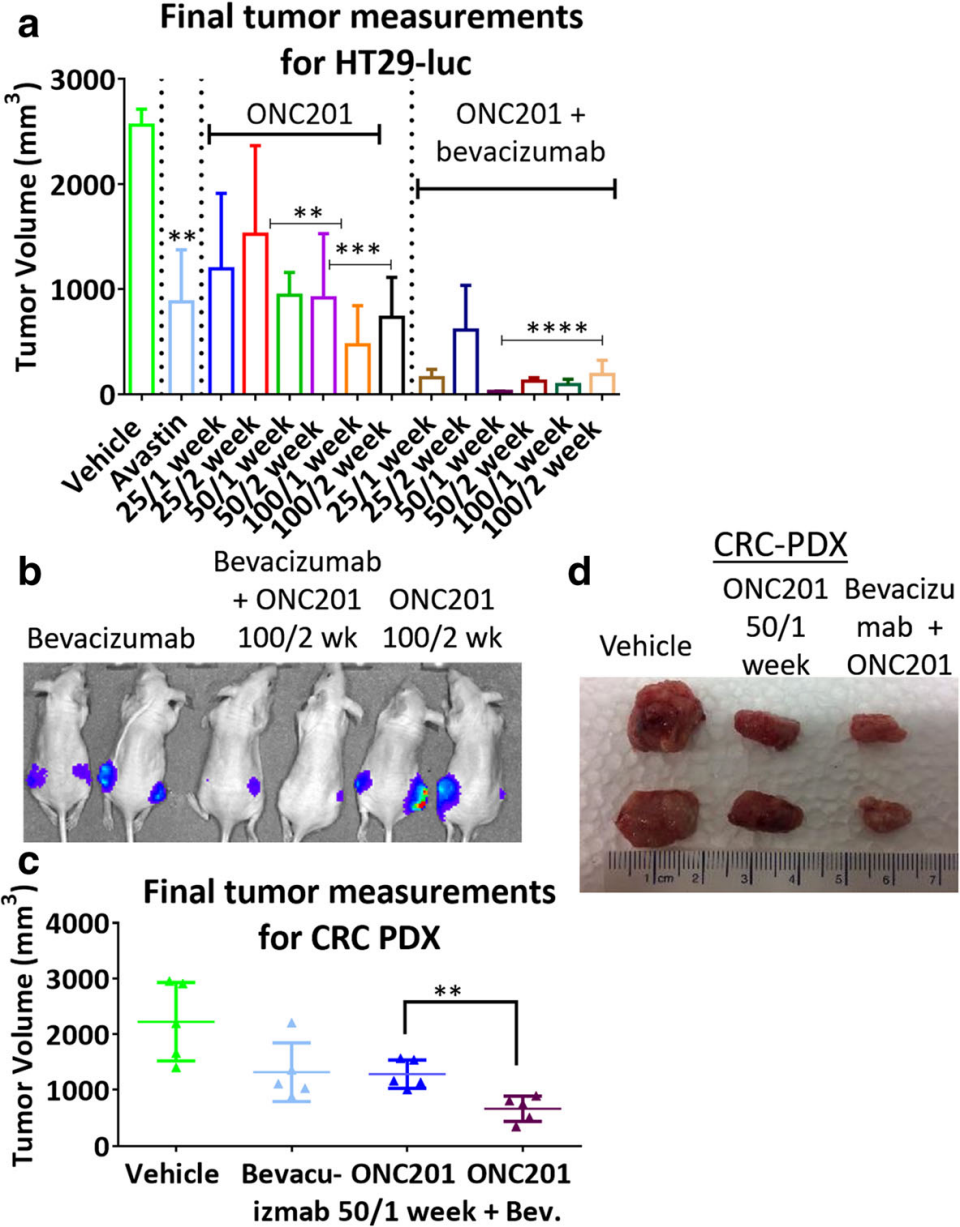
ONC201 acts in combination with bevacizumab to significantly impact colorectal cancer growth in vivo. **a** Final tumor measurements of HT29 human cell colorectal tumors in combination with ONC201 and bevacizumab at the indicated doses after 4 weeks. **b** Representative fluorescent images. **c** Final tumor measurements of CRC PDX tumors in SCID mice after 5 weeks. **d** Representative image of PDX tumors. Bevacizumab is 5 mg/kg every 2 weeks. *N* = 5

ONC201 has been shown to be efficacious in colorectal xenografts [4]. However, some aggressive xenografts are more resistant to ONC201’s treatment. We screened for potential synergy between ONC201 and approved CRC-based therapies (Additional file 1: Figure S1). Our in vitro results suggested no major synergies with 5-fluorouracil, oxaliplatin or irinotecan although there may be some additive effects. We further sought to investigate the combination of ONC201 and bevacizumab (Avastin) since bevacizumab is an FDA-approved and relatively well-tolerated drug used to treat patients with advanced colorectal cancer. Assessing for a potential synergy between ONC201 and bevacizumab in vivo, we observed that in the HT29 xenograft model, the combination of ONC201 and bevacizumab led to a significant reduction in tumor growth and development (Fig. 1a-b). Both bevacizumab alone and the combination led to no metastases forming during tumor development (Additional file 2: Figure S2). This combination was also synergistic against colorectal PDX tumors, and more potent in suppressing tumor growth as compared to ONC201 or bevacizumab alone. (Fig. 1c). The combination of ONC201 plus bevacizumab was also shown to be non-toxic (Additional file 3: Figure S3).

### ONC201 shows combinatorial efficacy with syngeneic colorectal tumors and mouse anti-angiogenic anti-VEGF or regorafenib in vivo

One limitation that has been noted over the years is that in preclinical models of human xenografts, tumor growth is rapid and not supported by vascular structures; particularly from a different species such as mouse host. Using humanized monoclonal antibodies also only attacks the human vasculature that was able to grow within the xenograft, and not within the host. Thus, tumor vessels within murine tumors implanted in syngeneic mouse models tend to be more prevalent and are able to grow within the tumor [31, 32]. To better demonstrate the relevance of VEGF inhibitors in a complete system, we chose to use a murine VEGF-A inhibitory antibody, a murine counterpart of bevacizumab, in syngeneic models injected with murine CRC.

**Fig. 2:**
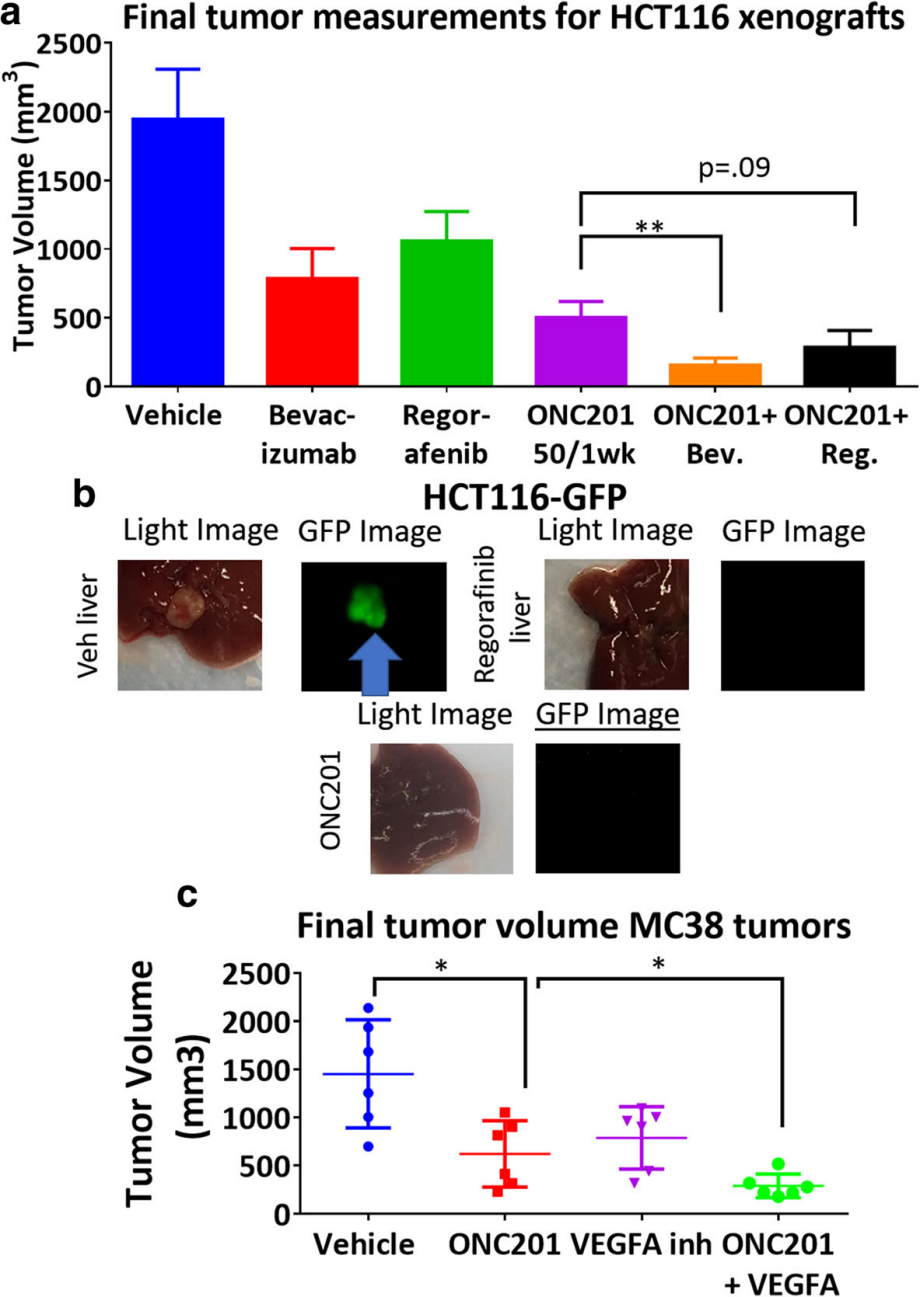
ONC201 acts in combination with murine VEGF inhibitors and anti-angiogenic agent regorafenib in vivo to suppress tumor growth. **a** Final tumor measurements of HCT116 xenografts with ONC201 or the indicated combinations after 3 weeks. **b** Light microscopy and fluorescent GFP images of metastases in vehicle, regorafenib, and ONC201 treated mice. **c** Relative tumor growth of MC38 tumors in C57/BL6 mice after 4 weeks. ONC201: 50 mg/kg every week. Regorafenib: 5 mg/kg daily. Bevacizumab: 5 mg/kg every other week. *N* = 5

To determine whether ONC201 can also synergize with other anti-angiogenic agents; we treated mice with regorafenib and a murine VEGF-A inhibitor in combination with ONC201. ONC201 significantly synergized with either bevacizumab or the murine anti-VEGF-A (Fig. 2). Although the combination of ONC201 with the less specific compound, regorafenib, was not as significant as compared to VEGF-inhibitors, regorafenib/ONC201 therapy still tended to be more potent than mono-agent therapy (Fig. 2a-b, Additional file 2: Figure S2). Similar to what was observed with bevacizumab, regorafenib in combination with low doses of ONC201, reduced the incidence of metastases as compared to low doses of ONC201 alone (Fig. 2c). These drug combinations were also found to be non-toxic in mice as seen by no notable significant side effects during treatment, no significant impact on serum blood chemistries, and a normal organ pathological analysis. (Additional file 3: Figure S3).

### ONC201 does not significantly impact VEGF levels or human vascular endothelial cell (HUVEC) growth in vitro and does not impact vascular growth in vivo

**Fig. 3:**
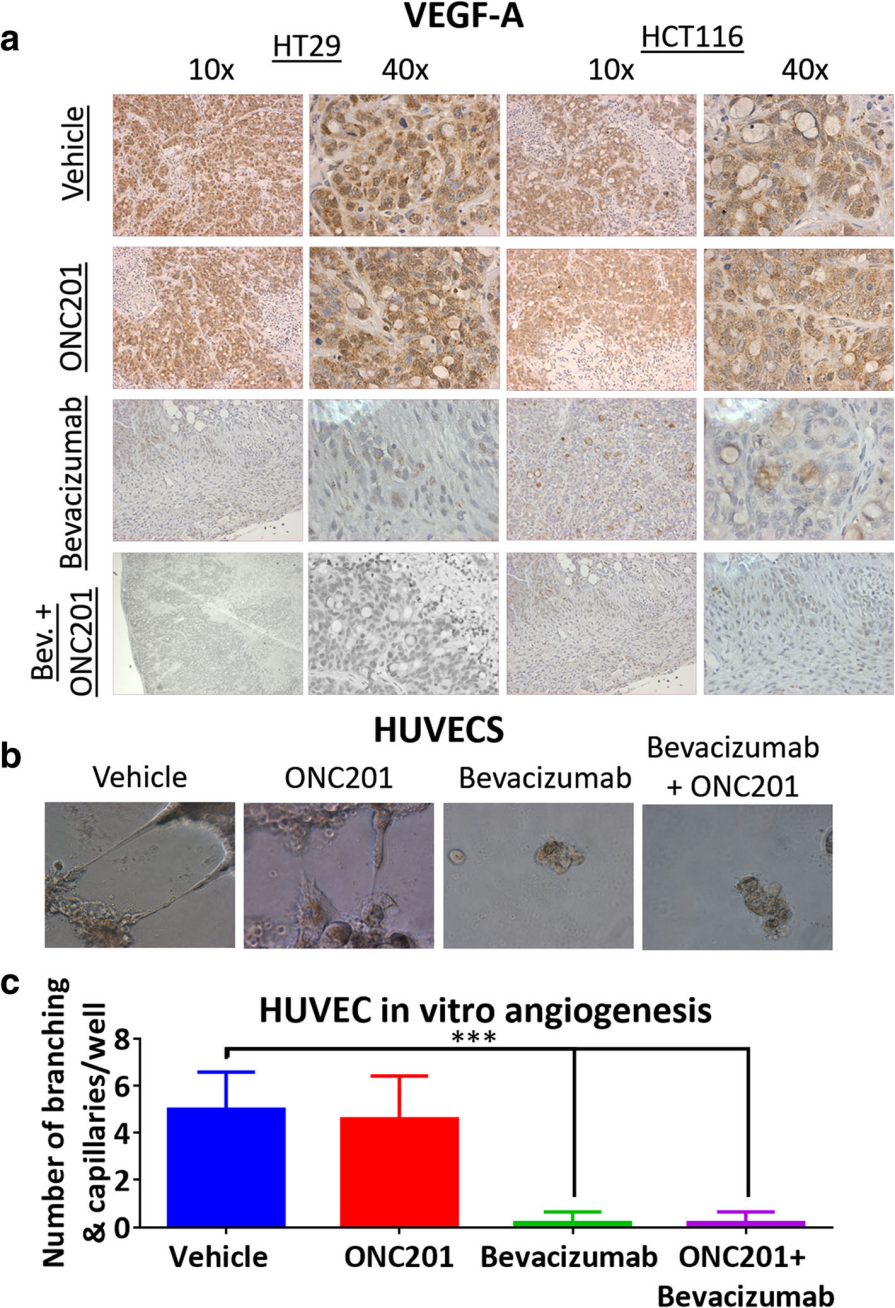
ONC201 does not impact VEGF expression in xenografts or HUVEC sprouting. **a** VEGF-A expression as detected by immunohistochemistry in HT29 and HCT116 CRC xenografts. **b** HUVEC representative images of sprouting from HUVECs grown on Matrigel. **c** Quantitation of HUVEC sprouting and branching after 12 h of drug treatment. In vivo: *n* = 5 ONC201 treatment dose was 50 mg/kg weekly. HUVECS *N* = 4, ONC201 treatment dose 5 μM, bevacizumab dose 5 mg/ml

To determine potential mechanism(s) of synergy between ONC201 and anti-VEGF therapy, we screened for effects of the combinational therapies with focus on mono-agent key mechanisms of actions. In our colorectal xenograft, VEGF expression does not appear to be significantly impacted by weekly ONC201 treatment (Fig. 3). Not surprisingly, in the presence of bevacizumab, as an inhibitory VEGF monoclonal antibody, very little VEGF expression was observed (as bevacizumab masks VEGF detection by anti-VEGF antibodies). The combinational therapy also showed significantly less VEGF expression than the vehicle and ONC201 treatment (Fig. 3a, Additional file 4: Figure S4). We tested the effect of ONC201 on HUVEC sprouting and growth. While ONC201 did not impact the amount of HUVEC sprouting on Matrigel, bevacizumab and the ONC201-bevacizumab combination did inhibit HUVEC sprouting. (Fig. 3b-c). Interestingly, regorafenib alone or in combination did not impact HUVEC sprouting (Additional file 5: Figure S5a–b).

**Fig. 4:**
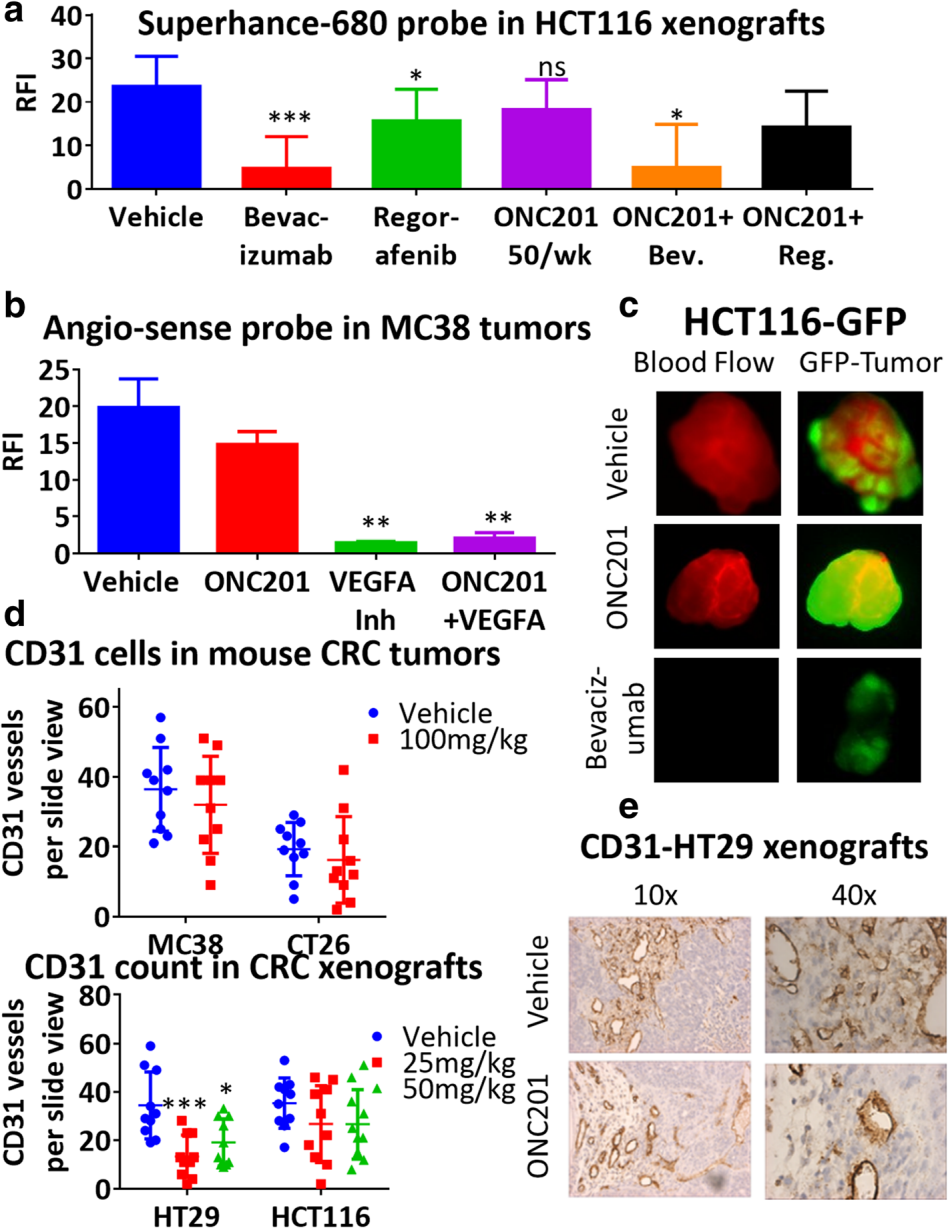
ONC201 does not inhibit vascular growth in vivo. Relative fluorescent intensity from (**a**) Superhance 680 probe in HCT116 xenografts and (**b**) Angio-sense probe in MC38 tumors after 4 weeks. **c** Representative images of HCT116 GFP tumors. CD31+ cell count per 20× slide view for (**d**) Syngeneic models CT26 and MC38 and E) Human xenograft HT29 and MC38. **e** Representative CD31 image for HT28 xenografts. For all in vivo experiments, *N* = 5. RFI was measured using image j and calculated for (tumor-background)/tumor area. CD31 staining was performed on multiple slides per tumor for tumors of similar size. (**e**)CD31-HT29 xenografts

We assessed the impact of the ONC201-bevacizumab combination therapy on tumor vascularization in vivo by performing histopathological analyses along with in vivo imaging. There have been several advancements in non-invasive imaging for monitoring vascularization within tumors. These methods can provide a means of detecting angiogenesis within the entire perimeter of a tumor and include PET studies [33]. For our purposes, we chose to use fluorescence imaging through the use of angiogenic probes. Bevacizumab and murine VEGF inhibitors decreased vascularization significantly, whereas ONC201 treatment did not impact blood flow in either mouse models. Further, ONC201 did not significantly increase the inhibition of vascularization caused by anti-angiogenic compounds. (Fig. 4a-c, Additional file 6: Figure S6).

The most commonly used method of assessing the success of anti-angiogenic therapies is measuring micro-vessel density (MVD) from biopsies taken before and after treatment, evaluating certain biomarkers including CD31 [34]. However, measurement of MVD can be problematic since blocking angiogenesis can lead to reduction of tumor growth and can affect the change in MVD. As an alternative, CD31 levels within tumors of similar size can be scored [35]. Therefore, we measured CD31 in tumors of comparable size after long-term treatment or measured CD31 after short treatment before tumor reduction could occur. ONC201 did not appear to impact CD31 levels within two human xenografts and two murine colorectal tumors (Fig. 4d-e, Additional files 7: Figure S7 and 8: Figure S8).

### Bevacizumab and regorafenib do not impede ONC201’s TRAIL serum induction levels but do augment apoptotic fragment M30 levels

**Fig. 5:**
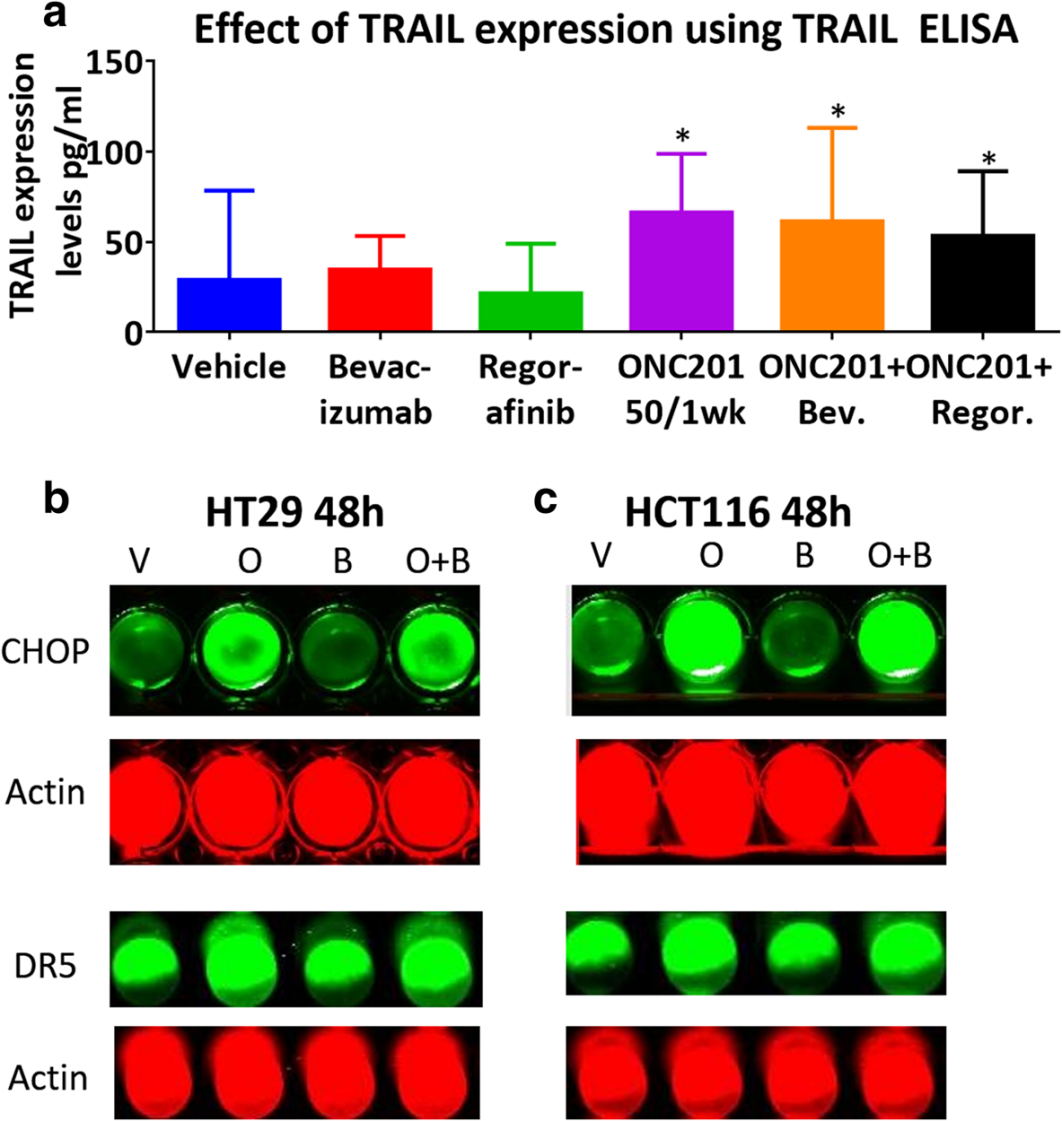
Bevacizumab and regorafenib do not appear to impact ONC201’s key mechanisms of cell death. **a** Serum TRAIL for TRAIL ELISA taken at week 3 in HCT116 xenograft-bearing athymic nude mice. *N* = 5. CHOP and DR5 protein in (**b**) HT29 and (**c**) HCT116 cells from live cell imaging using CHOP-800, DR5–800, and Actin-700 on LiCor Odyssey. Cells treated for 48 h. ONC201 dose was 5 μM. Bevacizumab dose was 1 mg/ml

In order to determine the mechanism of synergy between ONC201 and bevacizumab, we explored whether bevacizumab might promote ONC201-induced TRAIL expression (4) or ISR activation (5). We found that bevacizumab and regorafenib do not impact ONC201’s ability to induce serum TRAIL (Fig. 5a, Additional file 9: Figure S9) or CHOP/DR5 (Fig. 5b-c, Additional file 5: Figure S5 c) expression.

**Fig. 6:**
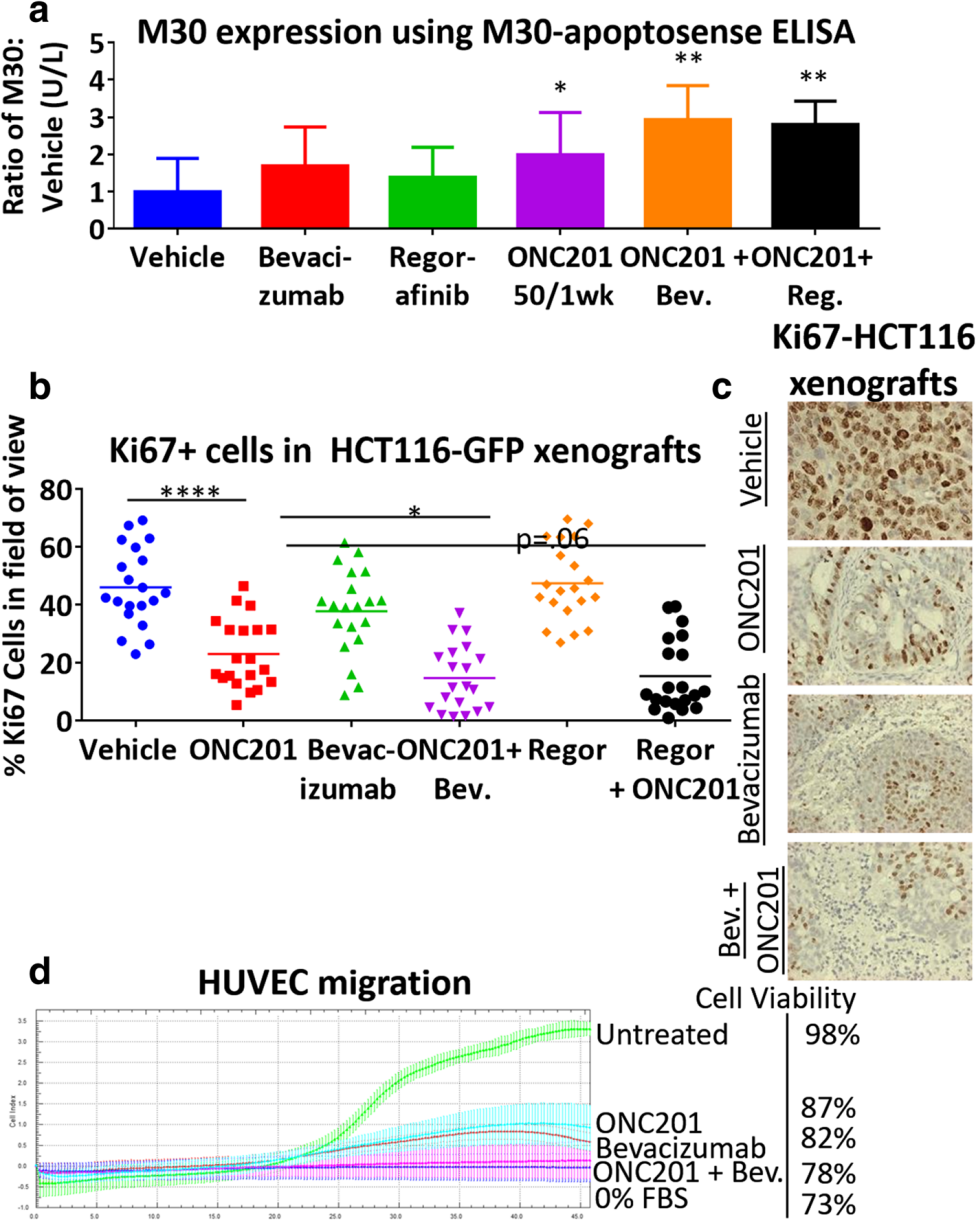
ONC201 and VEGF inhibitors act synergistically to impact apoptosis, growth arrest, and migration. **a** M30 expression from M30-apoptosense ELISA taken at week 3 in HCT116 xenograft-bearing athymic nude mice. *N* = 5. **b** Representative image and (**c**) quantification of Ki67+ cells in HT29 xenografts of ONC201, bevacizumab, and ONC201 + bevacizumab in combination. *N* = 20 slides, 5 per tumor. **d** Migration assay using xCelligence of HUVECS migrating to FBS + VEGF with +/− ONC201 treatment in upper chamber and +/− bevacizumab in lower chamber. *N* = 4

**Fig. 7:**
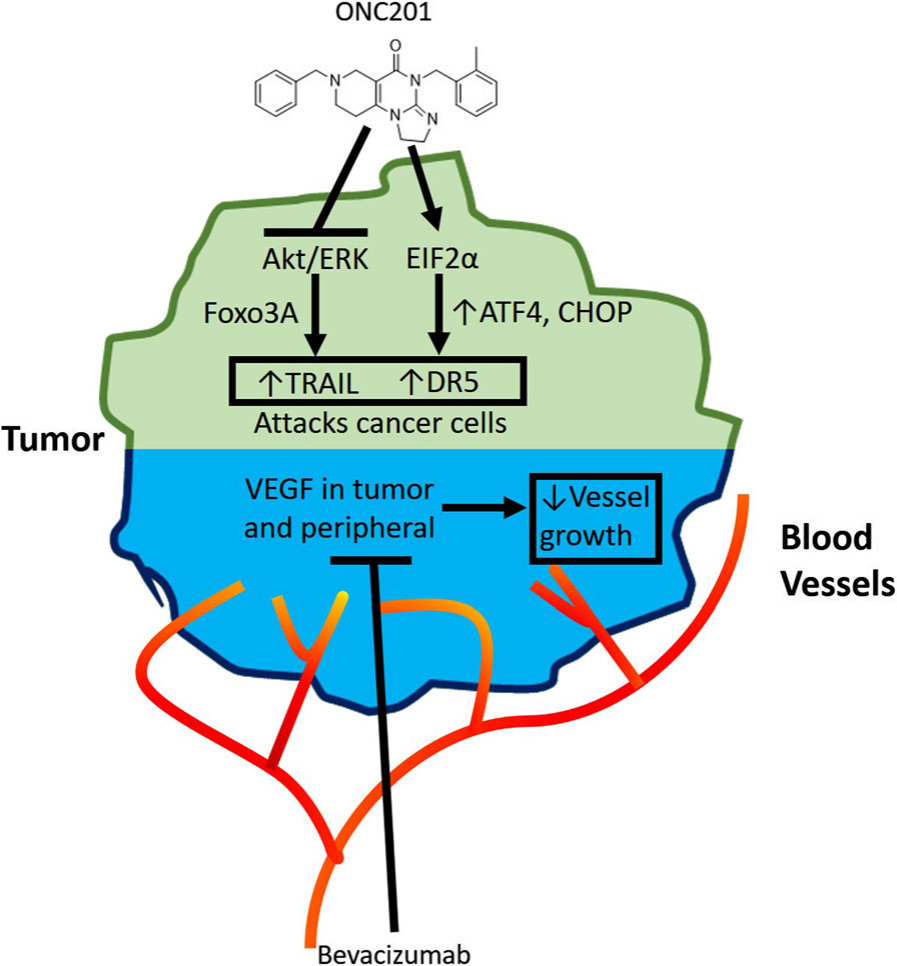
Schematic representation of a novel combination therapy for advanced colorectal cancer. Schematic representation of a novel combination therapy using ONC201 (top) and bevacizumab (bottom). The treatments invoke complementary yet non-overlapping independent mechanisms to increase tumor cell death and inhibit proliferation. (Top) ONC201 mechanism of action as defined by upregulation of TRAIL and DR5. (Bottom) Bevacizumab’s mechanism of action as defined by inhibition of VEGF in tumor and peripheral cells, leading to a decrease in blood vessel development

We assessed whether the ONC201-bevacizumab combination increases apoptosis and/or inhibits proliferation by analyzing the apoptotic caspase cleavage product M30 and Ki67 expression as a proliferation marker, respectively. A slight increase in M30 serum levels was observed in tumor tissues from combination-treated mice. In addition, a significant decrease in Ki67 was observed in mice treated with either bevacizumab-ONC2091 or regorafenib-ONC201 combination as compared to single agents (Fig. 6a-c, Additional files 10: Figure S10 and 11: Figure S11). Furthermore, ONC201 and bevacizumab in combination significantly inhibited HUVEC migration at low doses that did not induce toxicity compared to monotherapy treatment (Fig. 6d). This data supports the idea that ONC201 and bevacizumab mechanistically act independently of one another, but do increase each other’s ability to promote tumor apoptosis and decrease tumor cell growth (Fig. 7).

## Discussion

While as a single agent, ONC201 can be efficacious at a dose of 50 mg/kg in vivo, we have demonstrated here that in combination with the clinically approved agents bevacizumab or regorafenib, ONC201 can significantly reduce tumor growth in colorectal xenografts. Based on significant effects on tumor growth over a 4-week period, the combination of ONC201 with anti-VEGF therapies seems promising as compared to single agent therapies. Given the common use of VEGF inhibitors for combinational therapies in the clinic for CRC patients, this may be advantageous. Further, while bevacizumab and regorafenib are potent at preventing metastases from forming in mice, one might expect additive effects with the ONC201 therapies in the clinic, which should be addressed in clinical trials.

The success of ONC201 combination with murine VEGF inhibitors in syngeneic models was an important finding to demonstrate that VEGF therapies act both systemically on the host and locally within the tumor. In a direct comparison of efficacy, bevacizumab was far superior in combination with ONC201 than that or regorafenib. Further, the lack of toxicity observed in the ONC201-bevacizumab combination-treated mice, compared to the effects seen in the regorafenib-ONC201 combination mice, supports the conclusion that ONC201-bevacizumab combination would be favored for initial pursuit in the clinic.

As shown here, ONC201 has been demonstrated to not significantly impact VEGF, CD31+ cells, or angiogenesis. Further, VEGF-based compounds appear to not affect ONC201’s ability to induce TRAIL, CHOP, or DR5 levels; suggesting that ONC201 and anti-angiogenic agents mechanistically act independently of one another. Importantly, combinations of ONC201 with either clinically approved agent led to a greater increase in M30 and decrease in Ki67, along with a greater inhibition of HUVEC migration. This demonstrates that while acting through two separate mechanisms, both ONC201 and combination with either bevacizumab or regorafenib enhance tumor cell death and inhibit proliferation when used in combination. Based on our results, such a trial of ONC201 and bevacizumab in combination for the treatment of CRC would include biomarkers for each drug individually, and a biomarker selected for the effect of the drugs in combination, such as caspase-cleavage product M30. This is commonly used in dual-therapeutic treatments. One limitation is that we pursued in preclinical models only dosing at the same time, and other schedules should be pursued. Further, chemo-resistant tumors were not analyzed and need to be taken into consideration. Biomarker panels as discussed would aid in determining the optimal patient populations for this treatment.

## Conclusion

Our data here indicates that ONC201 and bevacizumab act through independent mechanisms; with ONC201 impacting TRAIL and DR5 pathways and bevacizumab acting as an anti-angiogenic agent. In combination, they increase tumor cell death markers and decrease proliferation at a higher rate than single agent alone without inducing any signs of drug toxicity. Our proposal of ONC201 and bevacizumab in combination provides a reasonable combination therapy with non-overlapping mechanisms of action, a strategy that historically has been associated with improved efficacy in the clinic. Moreover, this strategy could reduce the emergence of resistance cells in patients in the clinic and not subject patients to toxicities common with combination chemotherapies. Thus, ONC201 and bevacizumab could be a potent combination in the clinic for metastatic CRC patients and should be pursued further in clinical trials for patients with advanced or refractory metastatic disease.

## Additional file

Additional file 1: Figure S1. ONC201 and combination with CRC approved chemotherapeutics; CTG data. CTG data of HCT116 CRC cells treated with indicated doses and compounds for 72 hours. (PPTX 80 kb)
Additional file 2: Figure S2. Total number of metastases within liver and lung. A) Total number of metastases in HT29 tissues as seen in H&E slides by pathologist of lungs and liver. B) Total number of metastases seen in HCT116-GFP mice as seen by bioluminescent imaging and pathology of lungs and liver. (PPTX 75 kb)
Additional file 3: Figure S3. ONC201 and combination with bevacizumab are non-toxic in vivo. Blood chemistry panel results from mice treated with indicated drugs. Blood was collected by cardiac puncture at end of experiment. ONC201 50mg/kg weekly. Bevacizumab is 5 mg/kg every 2 weeks. Regorafenib: 5 mg/kg daily. N=3. (PPTX 98 kb)
Additional file 4: Figure S4. VEGF expression in HCT116 xenografts. Representative IHC staining of VEGF expression from mice treated with indicated drugs. Tumors harvested and placed in paraffin. ONC201: 50 mg/kg every week. Regorafenib: 5 mg/kg daily. N=5 tumors, minimum of 3 sections per tumor stained. (PPTX 195 kb)
Additional file 5: Figure S5. Analysis of regorafenib and ONC201 mechanism in combination. A) HUVEC representative images of sprouting from HUVECs grown on Matrigel. B) Quantitation of HUVEC sprouting and branching after 12 hours. C) HCT116 cells from live cell imaging using CHOP-800 and Actin-700 on LiCor Odyssey. Cells treated for 48 hours. ONC201: 5 μM. HUVECS N=4, ONC201 treatment 5 μM, Regorafenib 1 mg/ml. (PPTX 623 kb)
Additional file 6: Figure S6. Full imaging of Superhance blood flow and GFP from HCT116-GFP mice. Representative image using superhance 680 probe in HCT116 xenograft bearing athymic nude mice after 4 weeks. ONC201: 50 mg/kg every week. Regorafenib: 5 mg/kg daily. Bevacizumab: 5 mg/kg every other week. N=5. (PPTX 469 kb)
Additional file 7: Figure S7. CD31 expression in HT29 xenografts. Representative IHC staining of CD31 expression from mice treated with indicated drugs. Tumors harvested and placed in paraffin. ONC201: 50 mg/kg every week. Bevacizumab: 5 mg/kg every other week. N=5 tumors, minimum of 3 sections per tumor stained. (PPTX 184 kb)
Additional file 8: Figure S8. CD31 expression in MC38 CRC tumors. Representative IHC staining of CD31 expression from mice treated with indicated drugs. Tumors harvested and placed in paraffin. ONC201: 50 mg/kg every week. Anti VEGF-A (VEGF inhibitor): 10 μg twice weekly. N=6 tumors, minimum of 3 sections per tumor stained. (PPTX 199 kb)
Additional file 9: Figure S9. TRAIL and M30 ELISA standard curves. Standard curve of both TRAIL and M30 Elisa kits using the manufacturer’s instructions. (PPTX 66 kb)
Additional file 10: Figure S10. Ki67 staining of HCT116 xenografts. Representative IHC staining of Ki67 expression from mice treated with indicated drugs. Tumors harvested and placed in paraffin. ONC201: 50 mg/kg every week. Bevacizumab: 5 mg/kg every other week. Regorafenib: 5 mg/kg daily N=5 tumors, minimum of 3 sections per tumor stained. (PPTX 186 kb)
Additional file 11: Figure S11. Ki67 staining of HT29 xenografts. A) Representative IHC staining B) Quantitation using vectra and Inform analysis. Tumors harvested and placed in paraffin. ONC201: 50 mg/kg every week. Bevacizumab: 5 mg/kg every other week. N=5 tumors, minimum of 3 sections per tumor stained. (PPTX 1470 kb)

## Abbreviations

CRC: Colorectal cancer
DR5: Death-receptor 5
HUVEC: Human umbilical vein endothelial cells
ISR: Integrated stress response
PDX: Patient-derived xenograft
TRAIL: TNF-related apoptosis-inducing ligand
VEGF: Vascular endothelial growth factor
