# Correction: Anti-tumor effects of ONC201 in combination with VEGF-inhibitors significantly impacts colorectal cancer growth and survival in vivo through complementary non-overlapping mechanisms

**DOI:** 10.1186/s13046-024-03185-7

**Published:** 2024-09-11

**Authors:** Jessica Wagner, C. Leah Kline, Lanlan Zhou, Vladimir Khazak, Wafik S. El-Deiry

**Affiliations:** 1 Laboratory of Translational Oncology and Experimental Cancer Therapeutics, Molecular Therapeutics Program and Department of Hematology/Oncology, Fox Chase Cancer Center, Philadelphia, PA USA; 2 NexusPharma, Inc., Philadelphia, PA USA

**Correction: J Exp Clin Cancer Res 37, 11 (2018)**

**https://doi.org/10.1186/s13046-018-0671-0**

Following publication of the original article [1], the authors have been alerted to an error in Fig. 3A that shows a duplication of a histological image in two panels in the figure. This image duplication error in Fig. 3A was missed by all the authors and reviewers of the paper.

**Incorrect Fig. 3**

**Fig. 3:**
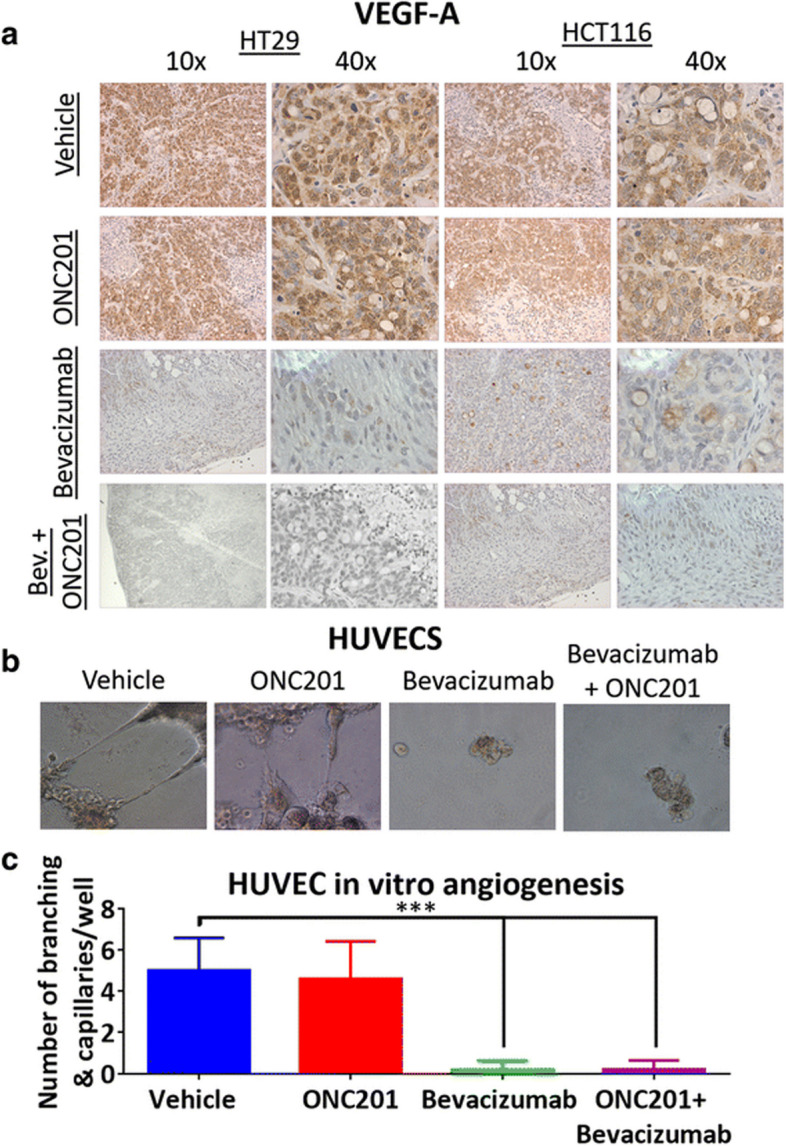
ONC201 does not impact VEGF expression in xenografts or HUVEC sprouting. **a** VEGF-A expression as detected by immunohistochemistry in HT29 and HCT116 CRC xenografts. **b** HUVEC representative images of sprouting from HUVECs grown on Matrigel. **c** Quantitation of HUVEC sprouting and branching after 12 h of drug treatment. In vivo: *n* = 5 ONC201 treatment dose was 50 mg/kg weekly. HUVECS *N* = 4, ONC201 treatment dose 5 μM, bevacizumab dose 5 mg/ml

**Correct Fig. 3**

**Fig. 3:**
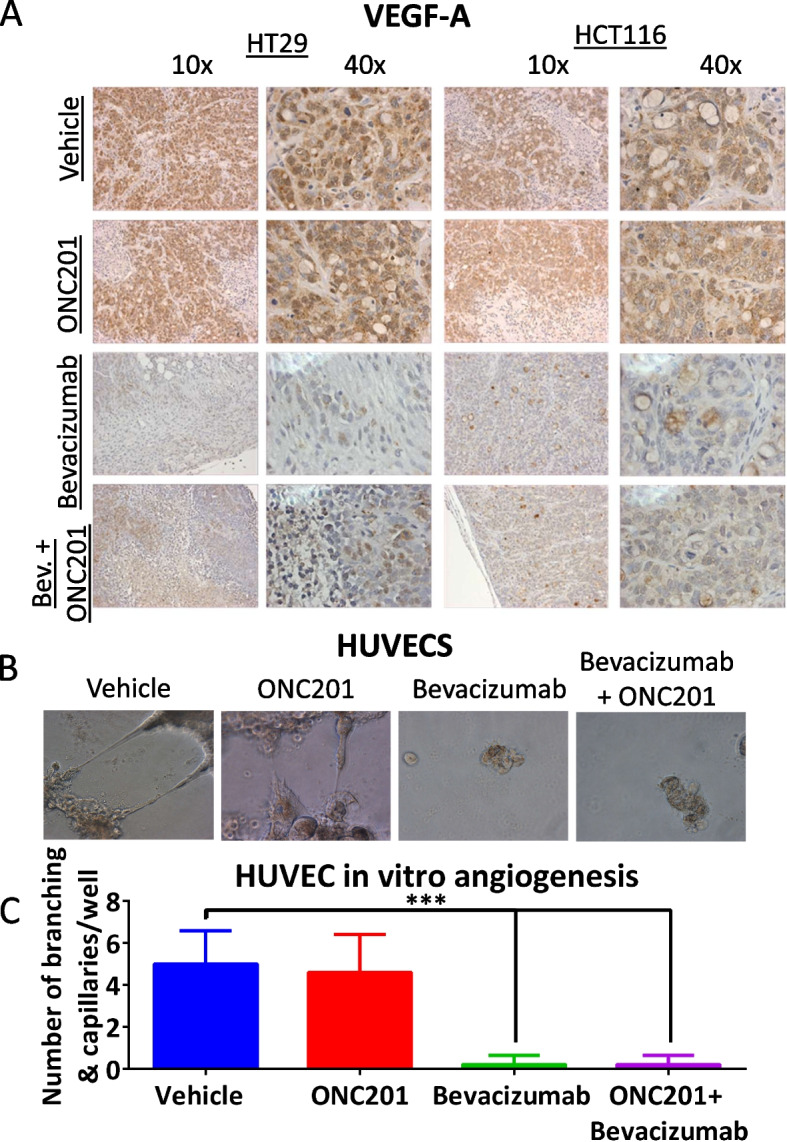
ONC201 does not impact VEGF expression in xenografts or HUVEC sprouting. **a** VEGF-A expression as detected by immunohistochemistry in HT29 and HCT116 CRC xenografts. **b** HUVEC representative images of sprouting from HUVECs grown on Matrigel. **c** Quantitation of HUVEC sprouting and branching after 12 h of drug treatment. In vivo: *n* = 5 ONC201 treatment dose was 50 mg/kg weekly. HUVECS *N* = 4, ONC201 treatment dose 5 μM, bevacizumab dose 5 mg/ml
