# Supplementary figures and images for: Anti-tumor effects of ONC201 in combination with VEGF-inhibitors significantly impacts colorectal cancer growth and survival in vivo through complementary non-overlapping mechanisms
Source: J Exp Clin Cancer Res. 2018 Jan 22;37:11. doi: 10.1186/s13046-018-0671-0 (PMC5778752; doi:10.1186/s13046-018-0671-0)

## Slide 1
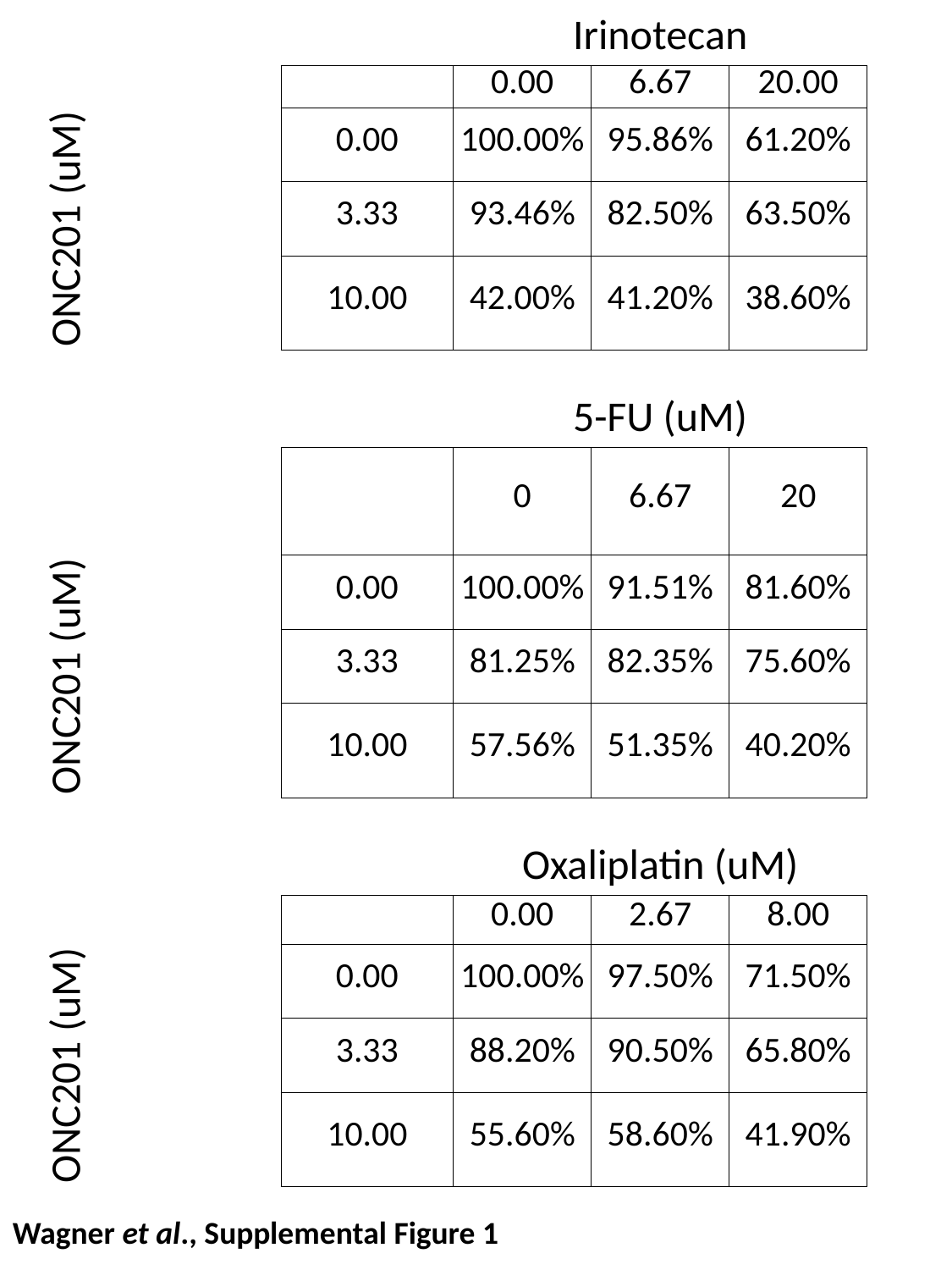

| | | Irinotecan | | |
| --- | --- | --- | --- | --- |
| | | 0.00 | 6.67 | 20.00 |
| ONC201 (uM) | 0.00 | 100.00% | 95.86% | 61.20% |
| | 3.33 | 93.46% | 82.50% | 63.50% |
| | 10.00 | 42.00% | 41.20% | 38.60% |
| | | | | |
| | | 5-FU (uM) | | |
| | | 0 | 6.67 | 20 |
| ONC201 (uM) | 0.00 | 100.00% | 91.51% | 81.60% |
| | 3.33 | 81.25% | 82.35% | 75.60% |
| | 10.00 | 57.56% | 51.35% | 40.20% |
| | | | | |
| | | Oxaliplatin (uM) | | |
| | | 0.00 | 2.67 | 8.00 |
| ONC201 (uM) | 0.00 | 100.00% | 97.50% | 71.50% |
| | 3.33 | 88.20% | 90.50% | 65.80% |
| | 10.00 | 55.60% | 58.60% | 41.90% |
Wagner et al., Supplemental Figure 1

Supplement: Additional file 1: Figure S1. — ONC201 and combination with CRC approved chemotherapeutics; CTG data. CTG data of HCT116 CRC cells treated with indicated doses and compounds for 72 hours. (PPTX 80 kb) [file 13046_2018_671_MOESM1_ESM.pptx]

## Slide 1
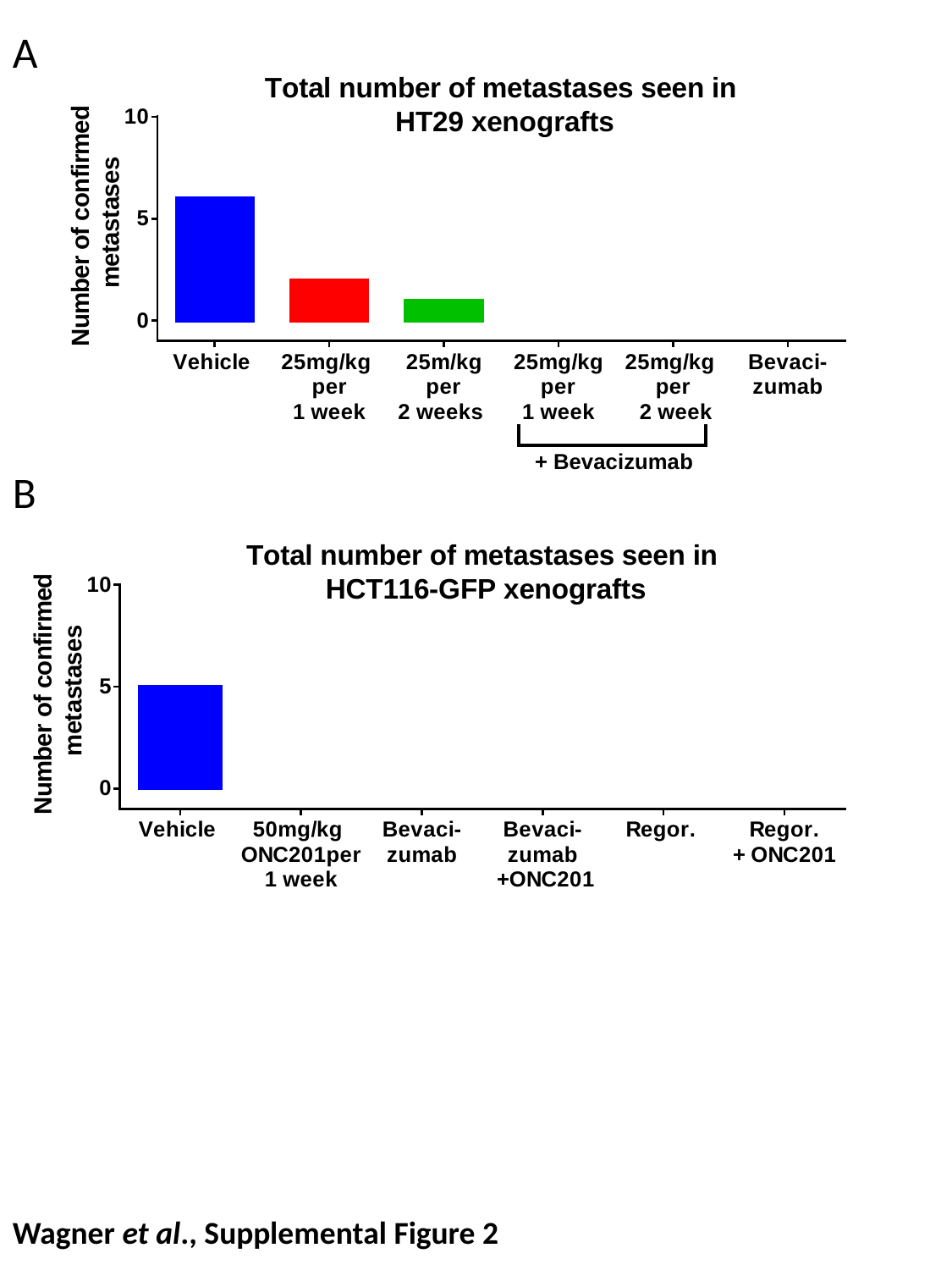

A
B
Wagner et al., Supplemental Figure 2

Supplement: Additional file 2: Figure S2. — Total number of metastases within liver and lung. A) Total number of metastases in HT29 tissues as seen in H&E slides by pathologist of lungs and liver. B) Total number of metastases seen in HCT116-GFP mice as seen by bioluminescent imaging and pathology of lungs and liver. (PPTX 75 kb) [file 13046_2018_671_MOESM2_ESM.pptx]

## Slide 1
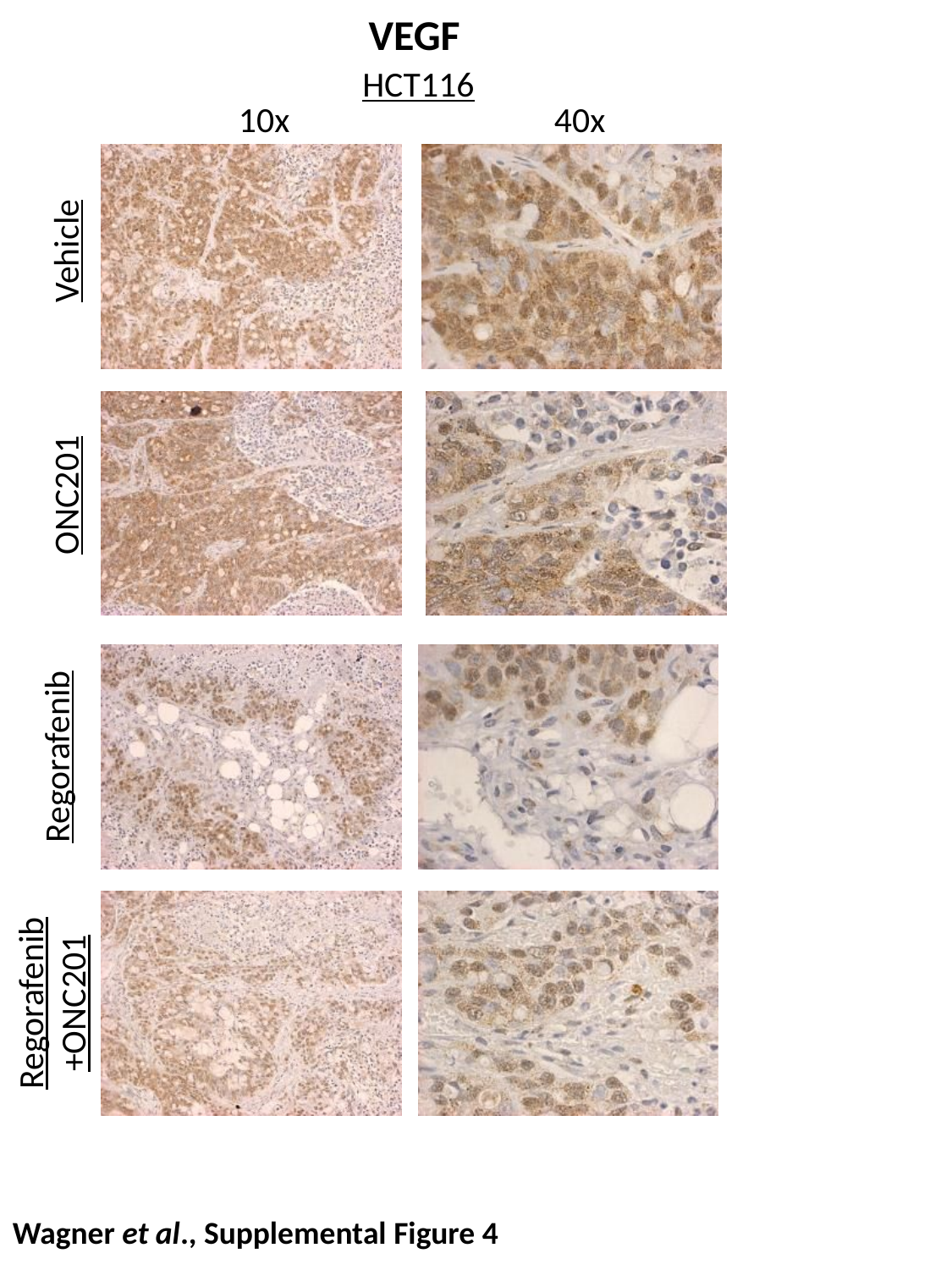

VEGF
HCT116
40x
10x
Vehicle
ONC201
Regorafenib
Regorafenib +ONC201
Wagner et al., Supplemental Figure 4

Supplement: Additional file 4: Figure S4. — VEGF expression in HCT116 xenografts. Representative IHC staining of VEGF expression from mice treated with indicated drugs. Tumors harvested and placed in paraffin. ONC201: 50 mg/kg every week. Regorafenib: 5 mg/kg daily. N=5 tumors, minimum of 3 sections per tumor stained. (PPTX 195 kb) [file 13046_2018_671_MOESM4_ESM.pptx]

## Slide 1
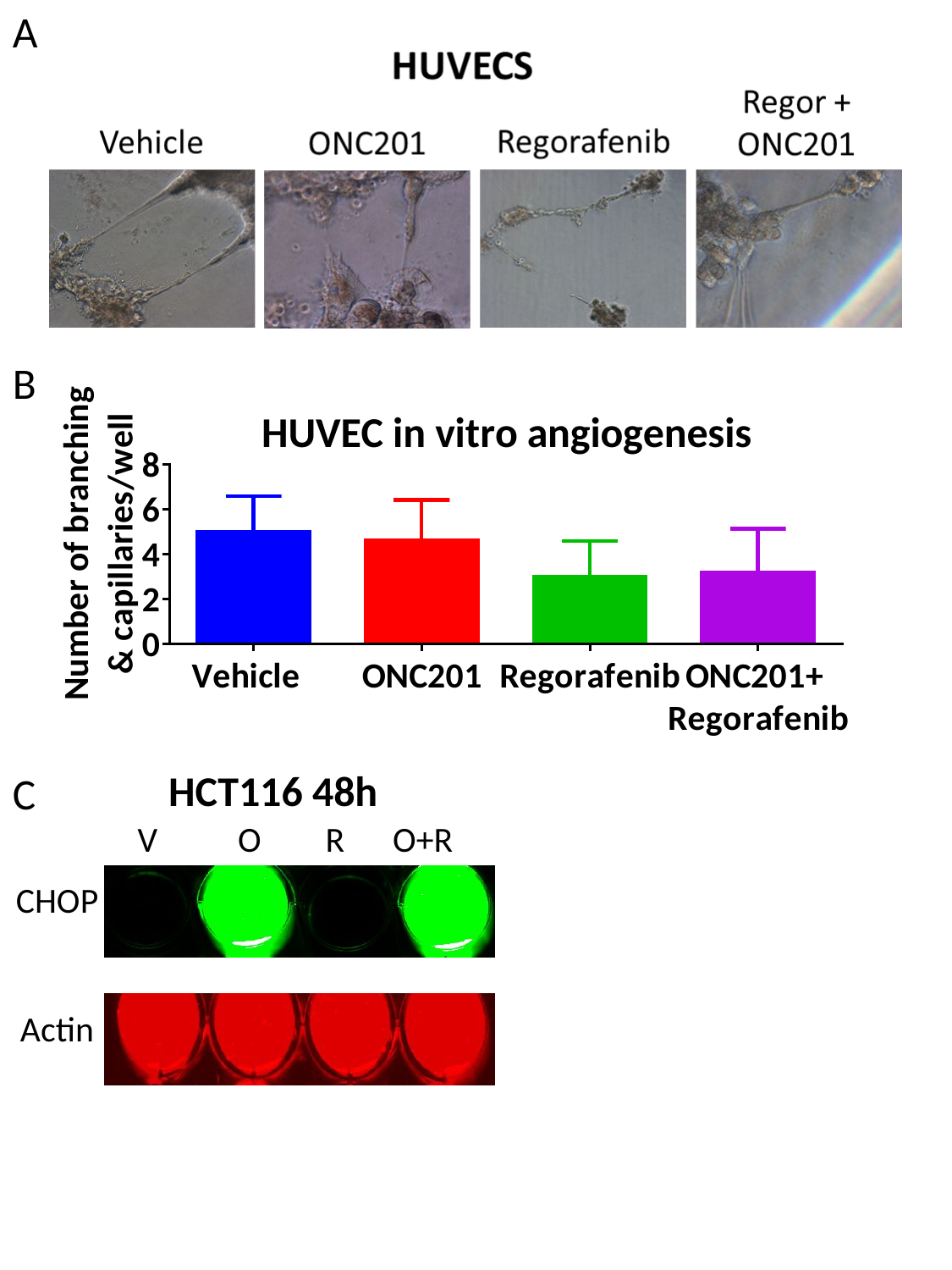

A
B
HCT116 48h
C
V O R O+R
CHOP
Actin

Supplement: Additional file 5: Figure S5. — Analysis of regorafenib and ONC201 mechanism in combination. A) HUVEC representative images of sprouting from HUVECs grown on Matrigel. B) Quantitation of HUVEC sprouting and branching after 12 hours. C) HCT116 cells from live cell imaging using CHOP-800 and Actin-700 on LiCor Odyssey. Cells treated for 48 hours. ONC201: 5 μM. HUVECS N=4, ONC201 treatment 5 μM, Regorafenib 1 mg/ml. (PPTX 623 kb) [file 13046_2018_671_MOESM5_ESM.pptx]

## Slide 1
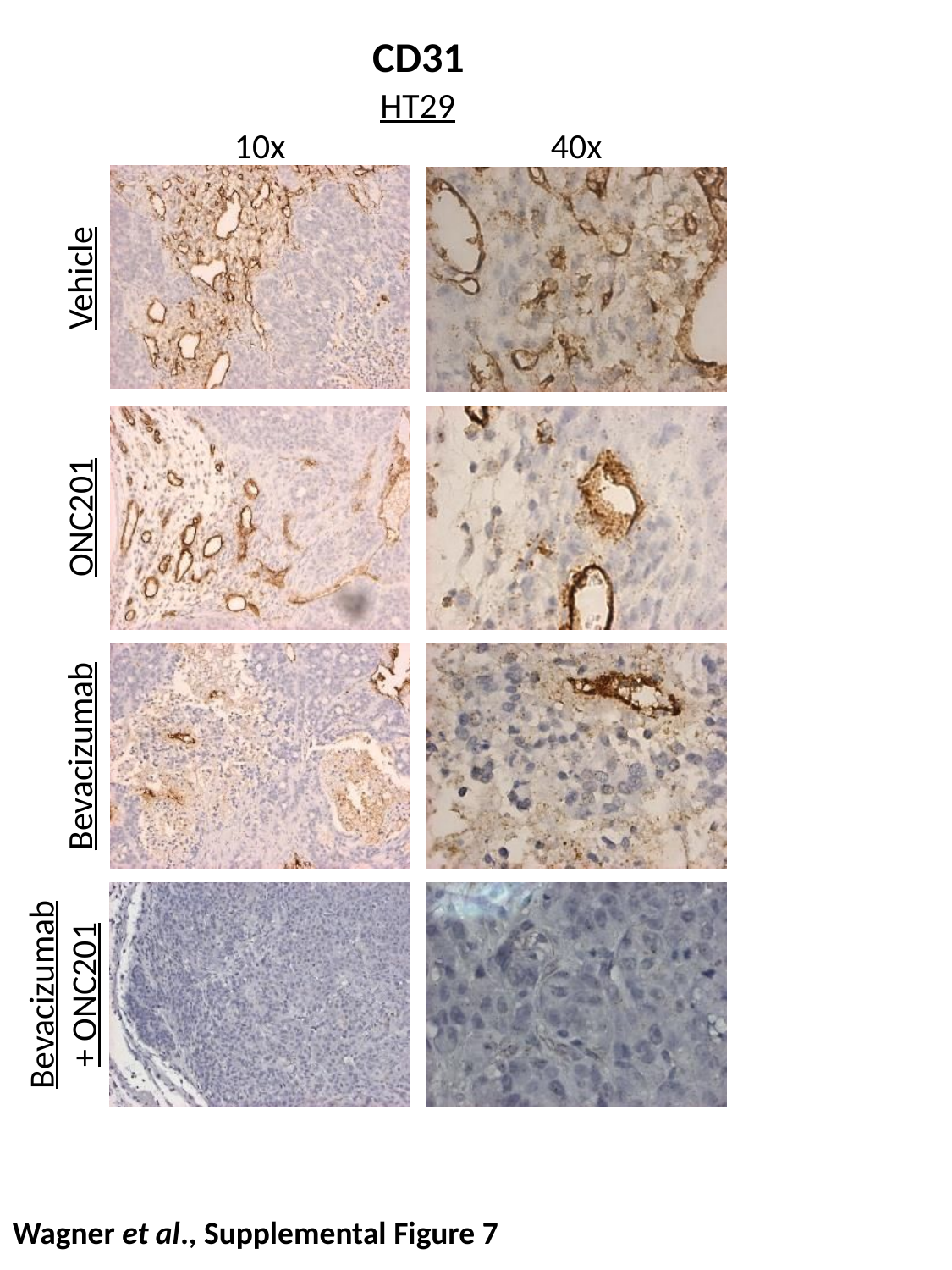

CD31
HT29
10x
40x
Vehicle
ONC201
Bevacizumab
Bevacizumab + ONC201
Wagner et al., Supplemental Figure 7

Supplement: Additional file 7: Figure S7. — CD31 expression in HT29 xenografts. Representative IHC staining of CD31 expression from mice treated with indicated drugs. Tumors harvested and placed in paraffin. ONC201: 50 mg/kg every week. Bevacizumab: 5 mg/kg every other week. N=5 tumors, minimum of 3 sections per tumor stained. (PPTX 184 kb) [file 13046_2018_671_MOESM7_ESM.pptx]

## Slide 1
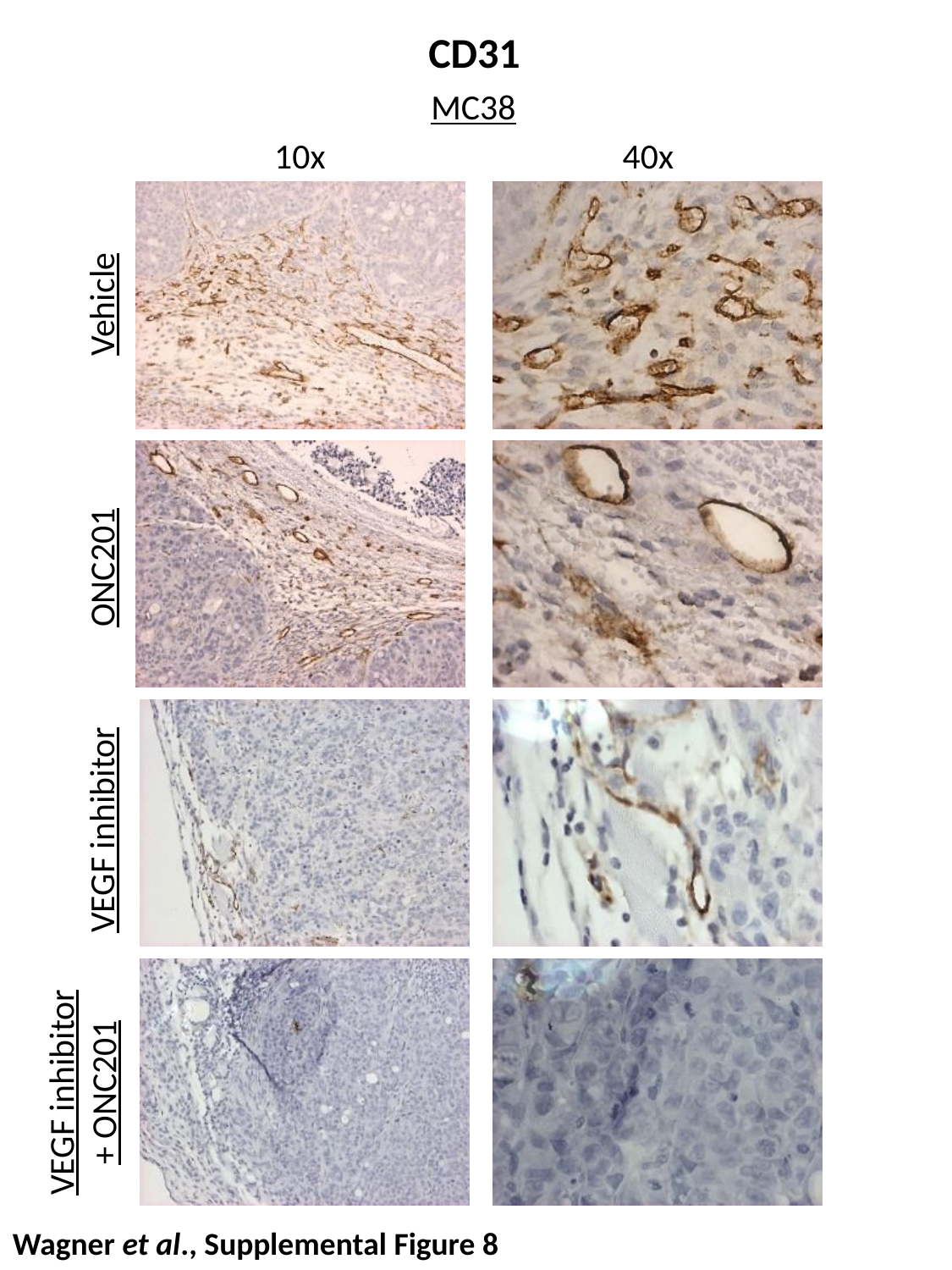

CD31
MC38
10x
40x
Vehicle
ONC201
VEGF inhibitor
VEGF inhibitor + ONC201
Wagner et al., Supplemental Figure 8

Supplement: Additional file 8: Figure S8. — CD31 expression in MC38 CRC tumors. Representative IHC staining of CD31 expression from mice treated with indicated drugs. Tumors harvested and placed in paraffin. ONC201: 50 mg/kg every week. Anti VEGF-A (VEGF inhibitor): 10 μg twice weekly. N=6 tumors, minimum of 3 sections per tumor stained. (PPTX 199 kb) [file 13046_2018_671_MOESM8_ESM.pptx]

## Slide 1
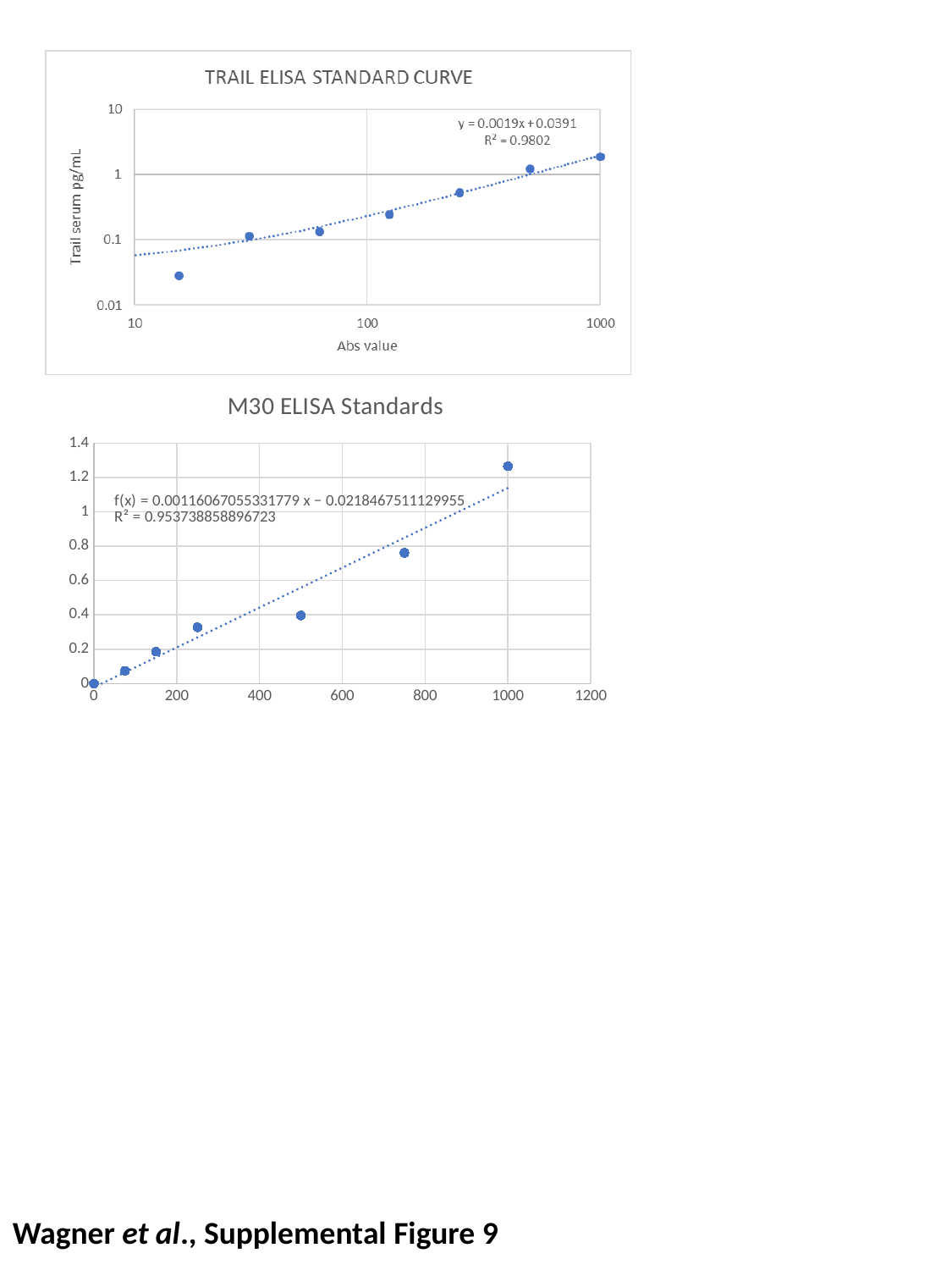

### Chart: M30 ELISA Standards
| Category | |
|---|---|Wagner et al., Supplemental Figure 9

Supplement: Additional file 9: Figure S9. — TRAIL and M30 ELISA standard curves. Standard curve of both TRAIL and M30 Elisa kits using the manufacturer’s instructions. (PPTX 66 kb) [file 13046_2018_671_MOESM9_ESM.pptx]

## Slide 1
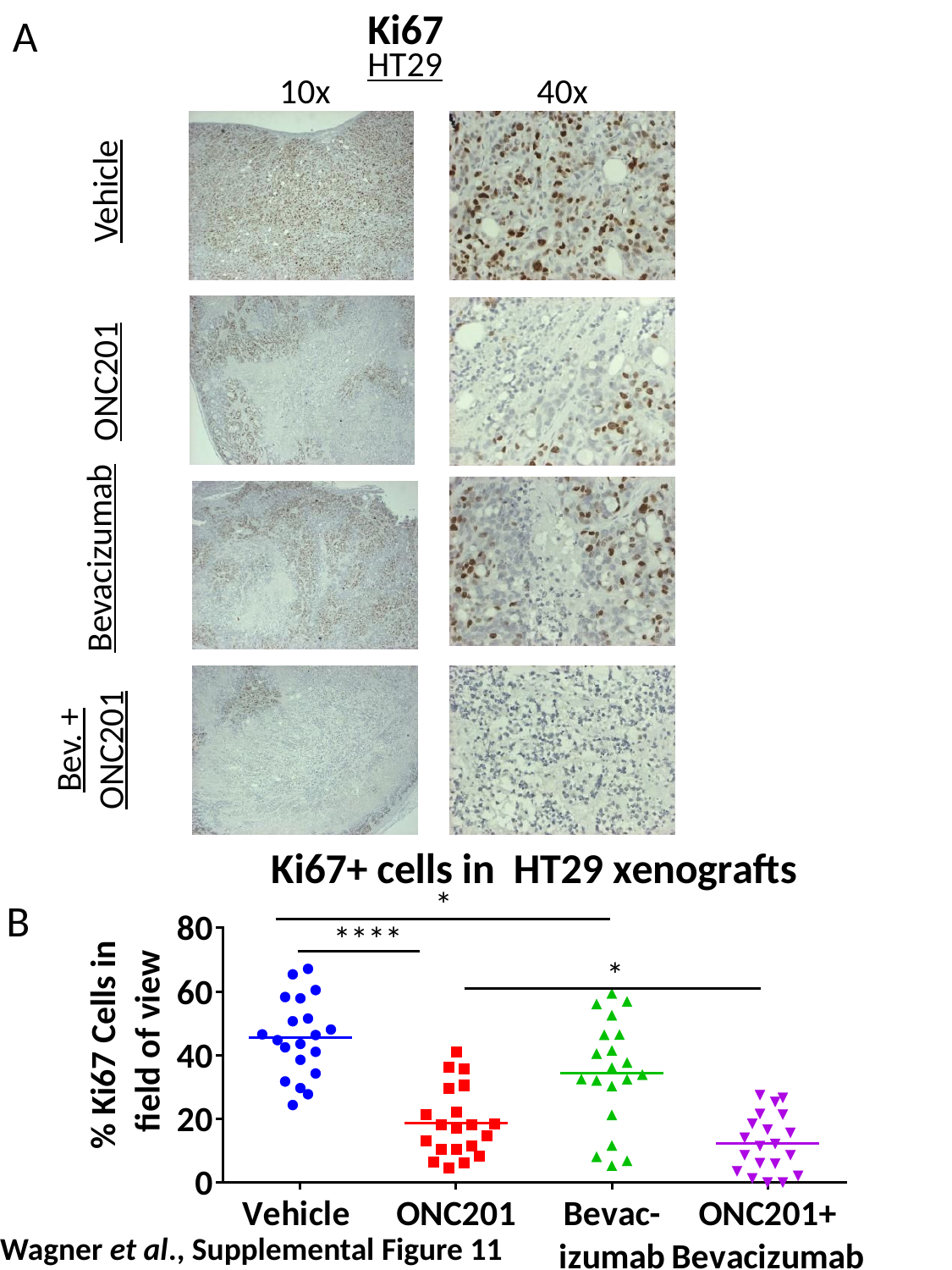

Ki67
A
HT29
10x
40x
Vehicle
ONC201
Bevacizumab
Bev. + ONC201
B
Wagner et al., Supplemental Figure 11

Supplement: Additional file 11: Figure S11. — Ki67 staining of HT29 xenografts. A) Representative IHC staining B) Quantitation using vectra and Inform analysis. Tumors harvested and placed in paraffin. ONC201: 50 mg/kg every week. Bevacizumab: 5 mg/kg every other week. N=5 tumors, minimum of 3 sections per tumor stained. (PPTX 1470 kb) [file 13046_2018_671_MOESM11_ESM.pptx]
