# Supplementary material for: Anti-tumor effects of ONC201 in combination with VEGF-inhibitors significantly impacts colorectal cancer growth and survival in vivo through complementary non-overlapping mechanisms
Source: J Exp Clin Cancer Res. 2018 Jan 22;37:11. doi: 10.1186/s13046-018-0671-0 (PMC5778752; doi:10.1186/s13046-018-0671-0)
Supplement: Additional file 3: Figure S3. — ONC201 and combination with bevacizumab are non-toxic in vivo. Blood chemistry panel results from mice treated with indicated drugs. Blood was collected by cardiac puncture at end of experiment. ONC201 50mg/kg weekly. Bevacizumab is 5 mg/kg every 2 weeks. Regorafenib: 5 mg/kg daily. N=3. (PPTX 98 kb) [file 13046_2018_671_MOESM3_ESM.pptx]

## Slide 1
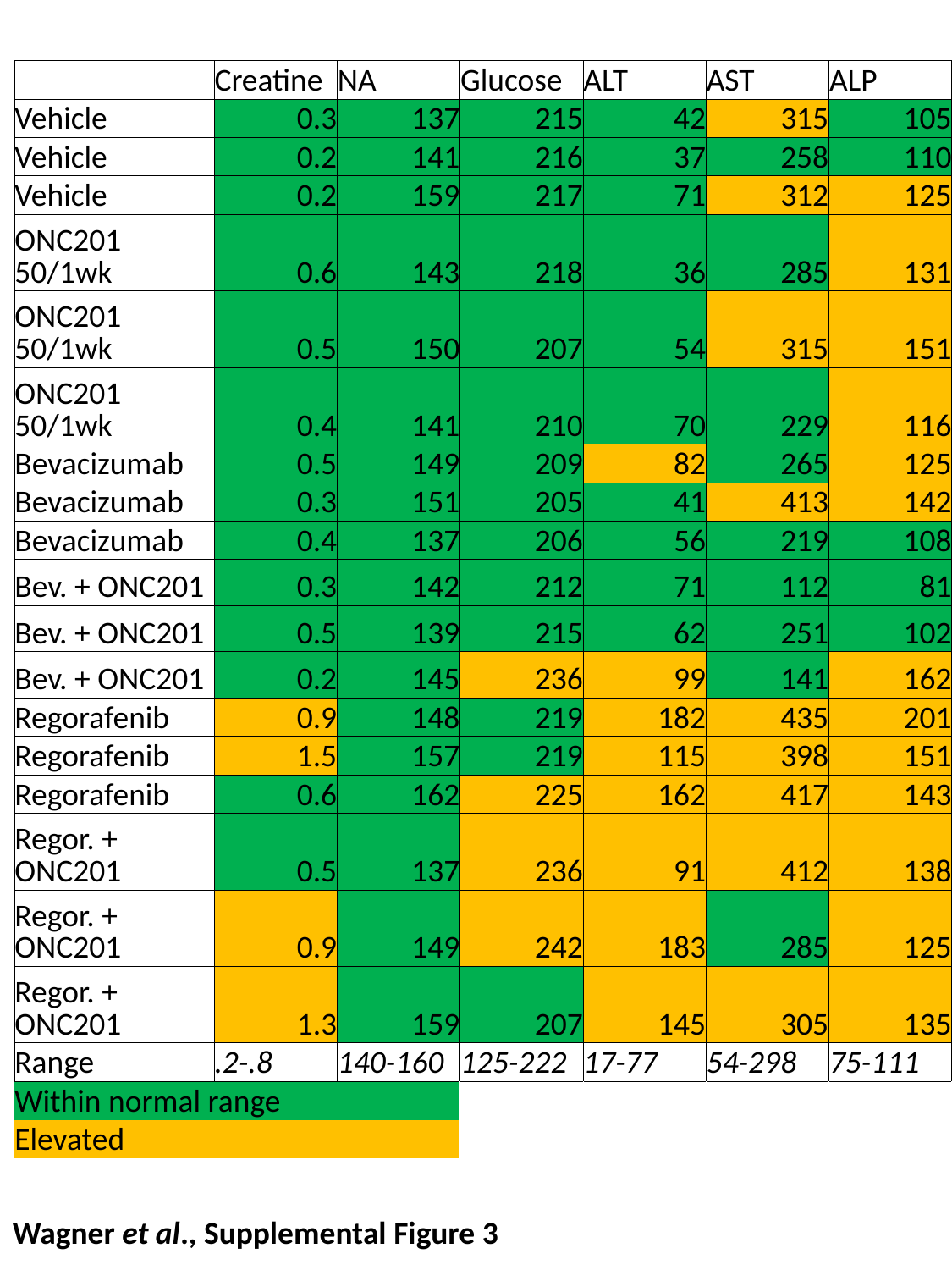

| | Creatine | NA | Glucose | ALT | AST | ALP |
| --- | --- | --- | --- | --- | --- | --- |
| Vehicle | 0.3 | 137 | 215 | 42 | 315 | 105 |
| Vehicle | 0.2 | 141 | 216 | 37 | 258 | 110 |
| Vehicle | 0.2 | 159 | 217 | 71 | 312 | 125 |
| ONC201 50/1wk | 0.6 | 143 | 218 | 36 | 285 | 131 |
| ONC201 50/1wk | 0.5 | 150 | 207 | 54 | 315 | 151 |
| ONC201 50/1wk | 0.4 | 141 | 210 | 70 | 229 | 116 |
| Bevacizumab | 0.5 | 149 | 209 | 82 | 265 | 125 |
| Bevacizumab | 0.3 | 151 | 205 | 41 | 413 | 142 |
| Bevacizumab | 0.4 | 137 | 206 | 56 | 219 | 108 |
| Bev. + ONC201 | 0.3 | 142 | 212 | 71 | 112 | 81 |
| Bev. + ONC201 | 0.5 | 139 | 215 | 62 | 251 | 102 |
| Bev. + ONC201 | 0.2 | 145 | 236 | 99 | 141 | 162 |
| Regorafenib | 0.9 | 148 | 219 | 182 | 435 | 201 |
| Regorafenib | 1.5 | 157 | 219 | 115 | 398 | 151 |
| Regorafenib | 0.6 | 162 | 225 | 162 | 417 | 143 |
| Regor. + ONC201 | 0.5 | 137 | 236 | 91 | 412 | 138 |
| Regor. + ONC201 | 0.9 | 149 | 242 | 183 | 285 | 125 |
| Regor. + ONC201 | 1.3 | 159 | 207 | 145 | 305 | 135 |
| Range | .2-.8 | 140-160 | 125-222 | 17-77 | 54-298 | 75-111 |
| Within normal range | | | | | | |
| Elevated | | | | | | |
Wagner et al., Supplemental Figure 3
