# Supplementary material for: Anti-tumor effects of ONC201 in combination with VEGF-inhibitors significantly impacts colorectal cancer growth and survival in vivo through complementary non-overlapping mechanisms
Source: J Exp Clin Cancer Res. 2018 Jan 22;37:11. doi: 10.1186/s13046-018-0671-0 (PMC5778752; doi:10.1186/s13046-018-0671-0)
Supplement: Additional file 6: Figure S6. — Full imaging of Superhance blood flow and GFP from HCT116-GFP mice. Representative image using superhance 680 probe in HCT116 xenograft bearing athymic nude mice after 4 weeks. ONC201: 50 mg/kg every week. Regorafenib: 5 mg/kg daily. Bevacizumab: 5 mg/kg every other week. N=5. (PPTX 469 kb) [file 13046_2018_671_MOESM6_ESM.pptx]

## Slide 1
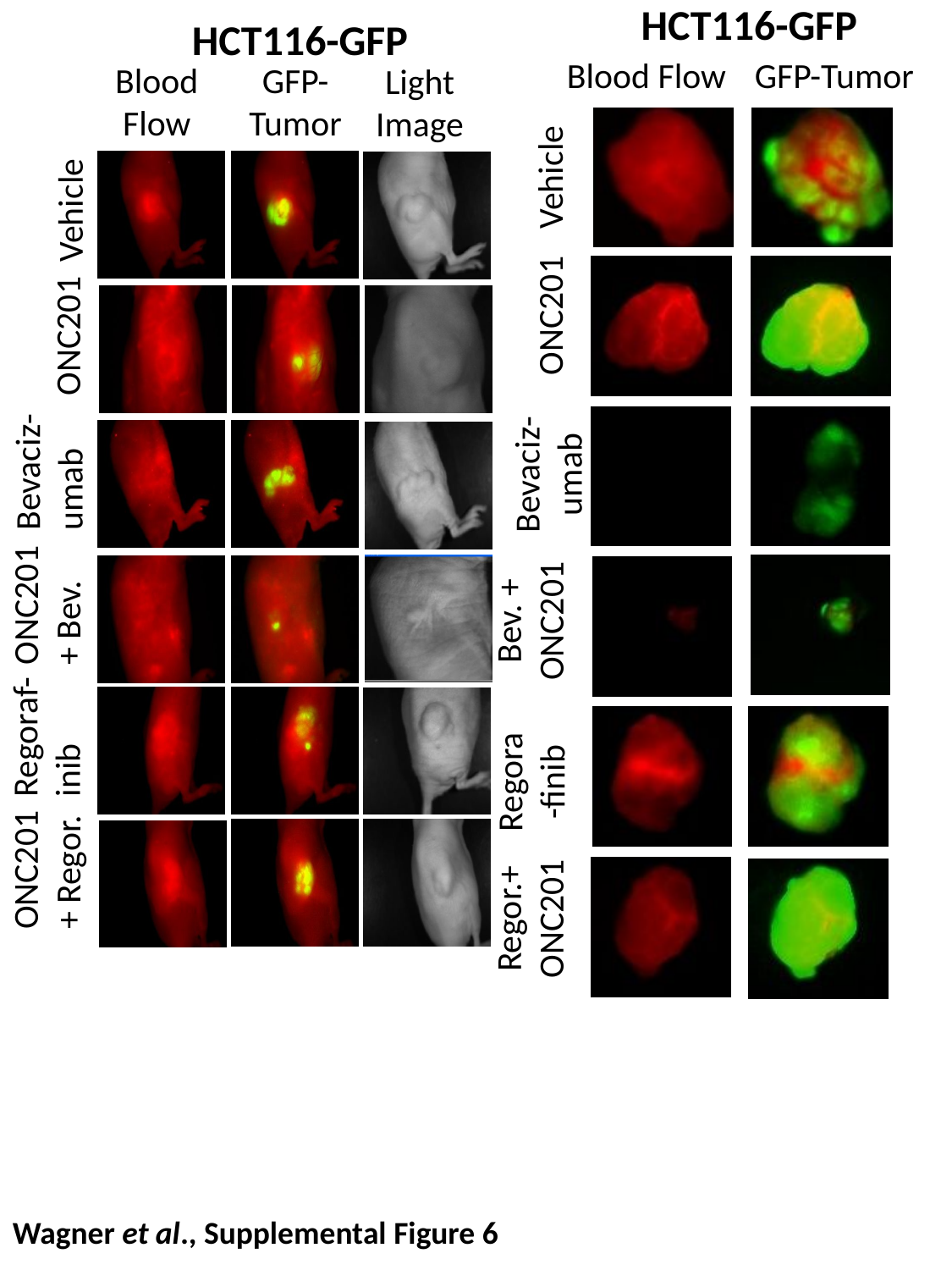

HCT116-GFP
HCT116-GFP
GFP-Tumor
Blood Flow
Light Image
Vehicle
ONC201
Bevaciz-umab
ONC201 + Bev.
Regoraf-inib
ONC201 + Regor.
Blood Flow
GFP-Tumor
Vehicle
ONC201
Bevaciz-umab
Bev. +
ONC201
Regora-finib
Regor.+ ONC201
Wagner et al., Supplemental Figure 6
