# Supplementary material for: Anti-tumor effects of ONC201 in combination with VEGF-inhibitors significantly impacts colorectal cancer growth and survival in vivo through complementary non-overlapping mechanisms
Source: J Exp Clin Cancer Res. 2018 Jan 22;37:11. doi: 10.1186/s13046-018-0671-0 (PMC5778752; doi:10.1186/s13046-018-0671-0)
Supplement: Additional file 10: Figure S10. — Ki67 staining of HCT116 xenografts. Representative IHC staining of Ki67 expression from mice treated with indicated drugs. Tumors harvested and placed in paraffin. ONC201: 50 mg/kg every week. Bevacizumab: 5 mg/kg every other week. Regorafenib: 5 mg/kg daily N=5 tumors, minimum of 3 sections per tumor stained. (PPTX 186 kb) [file 13046_2018_671_MOESM10_ESM.pptx]

## Slide 1
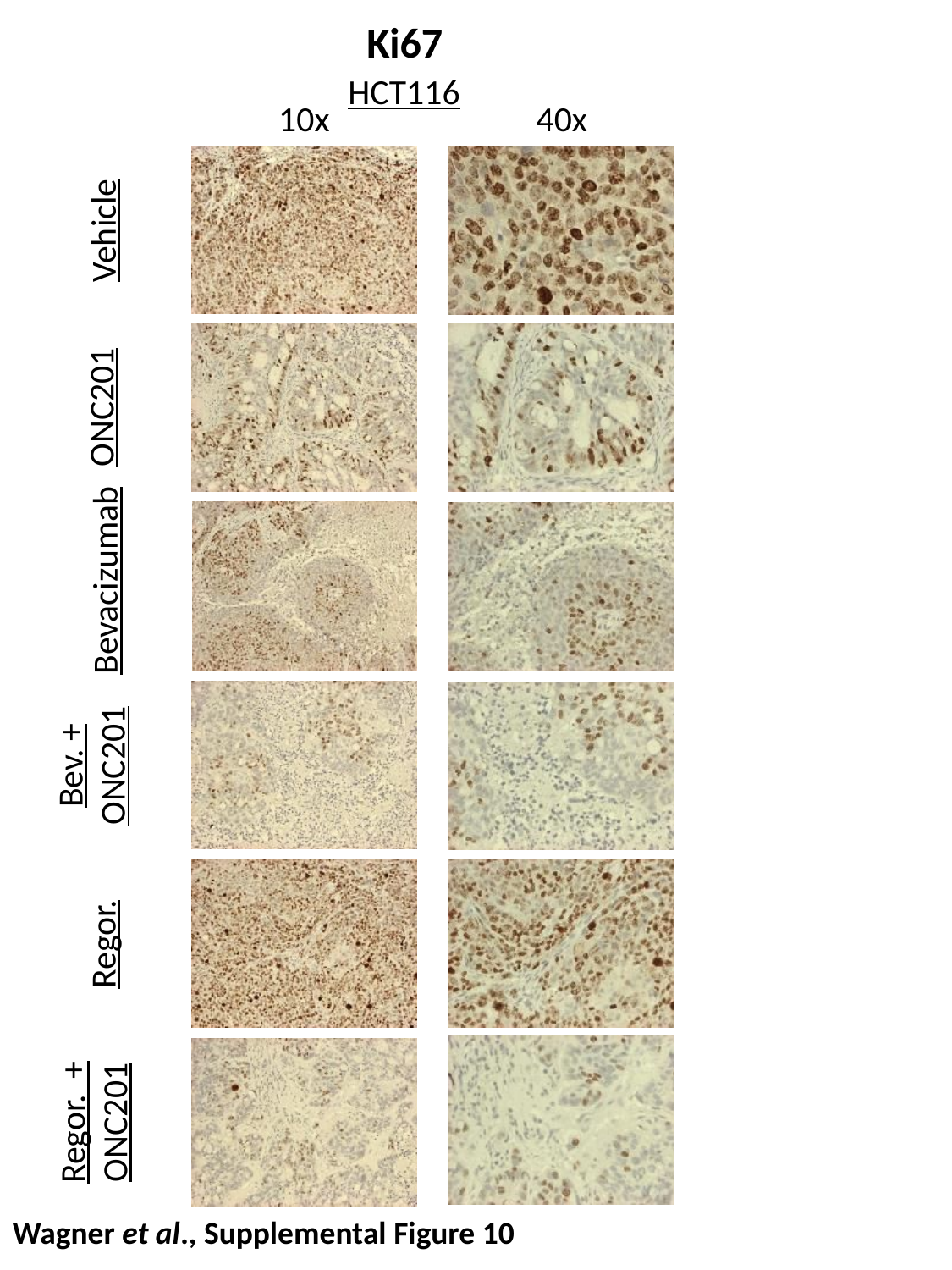

Ki67
HCT116
10x
40x
Vehicle
ONC201
Bevacizumab
Bev. + ONC201
Regor.
Regor. + ONC201
Wagner et al., Supplemental Figure 10
